# Human arm weight compensation in rehabilitation robotics: efficacy of three distinct methods

**DOI:** 10.1186/s12984-020-0644-3

**Published:** 2020-02-05

**Authors:** Fabian Just, Özhan Özen, Stefano Tortora, Verena Klamroth-Marganska, Robert Riener, Georg Rauter

**Affiliations:** 1 Sensory-Motor Systems (SMS) Lab, Institute of Robotics and Intelligent Systems (IRIS), Department of Health Sciences and Technology (D-HEST), ETH Zurich, Switzerland and Reharobotics Group, Spinal Cord Injury Center, Balgrist University Hospital, Medical Faculty, University of Zurich, Switzerland, Lengghalde 5, Zurich, 8092 Switzerland; 2 Motor Learning and Neurorehabilitation Laboratory, ARTORG Center for Biomedical Engineering Research, University of Bern, Freiburgstrasse 3, Bern, 3010 Switzerland; 3 IAS-Lab, Department of Information Engineering, University of Padova, via Giovanni Gradenigo 6a, Padova, 35131 Italy; 4 Institute of Occupational Therapy, School of Health Professions, Zurich University of Applied Sciences, Technikumstrasse 81, Winterthur, 8401 Switzerland; 5 BIROMED-Lab, Department of Biomedical Engineering, University of Basel, Düsternbrooker Weg 20, Allschwil, 4123 Switzerland

**Keywords:** Rehabilitation robotics, Arm weight compensation, EMG, Workspace assessment, Stroke

## Abstract

**Background:**

Arm weight compensation with rehabilitation robots for stroke patients has been successfully used to increase the active range of motion and reduce the effects of pathological muscle synergies. However, the differences in structure, performance, and control algorithms among the existing robotic platforms make it hard to effectively assess and compare human arm weight relief. In this paper, we introduce criteria for ideal arm weight compensation, and furthermore, we propose and analyze three distinct arm weight compensation methods (*Average*, *Full*, *Equilibrium*) in the arm rehabilitation exoskeleton ’ARMin’. The effect of the best performing method was validated in chronic stroke subjects to increase the active range of motion in three dimensional space.

**Methods:**

All three methods are based on arm models that are generalizable for use in different robotic devices and allow individualized adaptation to the subject by model parameters. The first method *Average* uses anthropometric tables to determine subject-specific parameters. The parameters for the second method *Full* are estimated based on force sensor data in predefined resting poses. The third method *Equilibrium* estimates parameters by optimizing an equilibrium of force/torque equations in a predefined resting pose. The parameters for all three methods were first determined and optimized for temporal and spatial estimation sensitivity. Then, the three methods were compared in a randomized single-center study with respect to the remaining electromyography (EMG) activity of 31 healthy participants who performed five arm poses covering the full range of motion with the exoskeleton robot. The best method was chosen for feasibility tests with three stroke patients. In detail, the influence of arm weight compensation on the three dimensional workspace was assessed by measuring of the horizontal workspace at three different height levels in stroke patients.

**Results:**

All three arm weight compensation methods reduced the mean EMG activity of healthy subjects to at least 49% compared with the no compensation reference. The *Equilibrium* method outperformed the *Average* and the *Full* methods with a highly significant reduction in mean EMG activity by 19% and 28% respectively. However, upon direct comparison, each method has its own individual advantages such as in set-up time, cost, or required technology. The horizontal workspace assessment in poststroke patients with the *Equilibrium* method revealed potential workspace size-dependence of arm height, while weight compensation helped maximize the workspace as much as possible.

**Conclusion:**

Different arm weight compensation methods were developed according to initially defined criteria. The methods were then analyzed with respect to their sensitivity and required technology. In general, weight compensation performance improved with the level of technology, but increased cost and calibration efforts. This study reports a systematic way to analyze the efficacy of different weight compensation methods using EMG. Additionally, the feasibility of the best method, *Equilibrium*, was shown by testing with three stroke patients. In this test, a height dependence of the workspace size also seemed to be present, which further highlights the importance of patient-specific weight compensation, particularly for training at different arm heights.

**Trial registration:**

ClinicalTrials.gov,NCT02720341. Registered 25 March 2016

## Background

### Human arm weight compensation in robotic rehabilitation

In the acute phase post-stroke, approximately 66*%*−80*%* of patients experience reduced arm function due to paresis and subsequent arm weakness [1, 2]. In chronic stroke patients abnormal synergies restrict the patient’s movement [3] and workspace [4] as a loss of independent joint control. In both acute and chronic patients, arm weight compensation can extend the patient’s workspace and, therefore, allow training of tasks that have higher relevance for activities of daily living [4–6]. The training of these tasks is according to the known “use it and improve it, or lose it” principle of neurorehabilitation, which is considered a key factor for successful rehabilitation [7]. Therefore, many rehabilitation robots with arm weight compensation functions have been developed. The common robot types that provide system-dependent arm weight compensation can be divided in gantry-based robots [8–10], passive exoskeletons [11, 12], actuated exoskeletons [4, 11, 13, 14], and actuated end-effector robots [5, 6, 15–18].

For actuated rehabilitation robots, assist-as-needed control strategies are also commonly used. These strategies not only support the patients along the movement direction, but also against the gravity in an indirect way [19, 20]. However, in this paper, we focus only on the arm weight compensation as an independent assistance dimension, since the support along movement direction may not always be desirable. Furthermore, there are adaptive control strategies that readjust assistance over time [21]. However, since arm weight is constant over time, we aim to estimate the arm weight parameters once in the beginning of the therapy session and not to adapt them during the therapy. From a literature review [8, 22] and our own experience, the following four requirements of ideal, generalizable weight compensation for robot-assisted training of activities of daily living were deduced: Freedom of movement, no additional disturbances, scalability, and applicability to other systems [13].

#### Freedom of movement

The degrees of freedom of the human arm joints should not be restricted by the robot. From the shoulder to the wrist joint, the human arm can be approximated by five degrees of freedom (shoulder horizontal abduction/adduction, shoulder elevation, shoulder internal/external rotation, elbow flexion/extension, forearm pronation/supination). Arm weight compensation should be provided in any pose without restricting or hindering any possible degree of freedom. However, most end-effector robots can restrict the user’s freedom of movement while providing arm weight compensation due to mechanical limitations and missing human joint angle information, e.g., [5, 6, 15–18].

#### No additional disturbances

With the exception of arm weight compensation, each robot should behave mechanically transparent during physical human-robot interactions [23]. Ideally, weight is a static force, and thus, the robot torques providing arm weight compensation should only depend on pose, arm weight, and arm length. Generally, end-effector robots support arm weight only at the end-effector without knowledge of the user’s arm joint angles, which can lead to over- or under-compensation of arm weight. In particular, systematic disturbances due to spring properties are present in passive exoskeletons [12], and unwanted horizontal forces due to the vertical sling attachments are present in gantry-based robots [8].

#### Scalability

A progressive reduction in arm weight compensation during rehabilitation therapy can lead to an increased active range of motion [24]. Furthermore, scalability of arm weight compensation can be used for assessment of arm weight-induced impairments [25]. Independent upper and lower arm weight scalability could allow more individualized assessments and therefore, tailored rehabilitation therapies for arm weight-induced impairments.

#### Applicability to other systems

The applicability of arm weight compensation methods to other systems is robot type-dependent. Exemplarily, a passive exoskeleton can entail mechanical spring-based arm weight compensation [12], which needs to be mechanically adapted for applications in different types of robots. Ideally, the method should be a software solution, that can be easily applied to a variety of rehabilitation robots with actuation.

### Evaluation of arm weight compensation efficacy

The highest arm weight compensation efficacy is reached through correctly compensating for gravity contributing to arm weight in every possible arm pose, i.e., overcompensation or undercompensation of arm weight leads to a lower arm weight compensation efficacy. Arm weight compensation has been proven to be an important factor for enabling patients to train for tasks that require longer reaching distances [5] and an increased workspace [6, 24–27]. Furthermore, improvements in arm weight compensation efficacy are expected to lead to an even greater increase in workspace for stroke patients [6]. However, most studies of arm weight compensation by rehabilitation robots have focused on the gains that stroke patients achieve in clinical scores rather than the provided arm weight compensation [28]. While gains in clinical scores are good indicators of performance development in general, it is difficult to assess the contribution of the provided weight compensation to these gains. Namely, the efficacy of weight compensation per se was not evaluated in parallel, i.e., how much unloading is effectively applied for a certain unloading condition and arm pose. Therefore, the results of clinical studies that evaluate weight compensation for one particular rehabilitation device are difficult to generalize to other devices, as the weight compensation performance might differ among devices. In summary, the efficacy of arm weight compensation in rehabilitation robotics has been rarely investigated [29]. However, several papers have investigated the efficacy of arm weight compensation through additional electromyography (EMG) measurements of relevant muscles [29–33]. For example, arm weight compensation decreased EMG activity during static holding [32] and reaching tasks [29–31], and the transfer of this effect to stroke patients was also shown [29]. However, static holding was performed in only one pose [32], not over the whole workspace to analyze pose-dependent effects of arm weight compensation. Furthermore, previous EMG signal analyses have mainly focused on individual muscle evaluations [29–31] instead of a combined evaluation of all relevant muscles with respect to their passive reference measurements as a score. Additional active EMG reference measurements to adapt to a subject’s specific behavior and physiology have not been a main focus [13]. Finally, the arm weight relief efficacies of different arm weight compensation methods have never been compared, even for use of the same device.

In this paper, we present three different arm weight compensation methods *Average*, *Full*, and *Equilibrium*. These arm weight compensation methods are based on arm models that are developed to address the presented four critical requirements. Each method has advantages and disadvantages regarding the technology used, hardware costs, and calibration effort/time. All three compensation methods are consecutively implemented and compared using the rehabilitation robot ARMin. This paper sequentially presents the following studies: First, the estimation results of the arm weight compensation methods are analyzed for spatial and temporal sensitivity. The efficacies of all three methods are subsequently tested with EMG measurements in healthy subjects. Finally, the most successful method is tested in stroke patients. During the assessment with stroke patients, the horizontal workspace is assessed at three different height levels to determine if there is a height-dependence of arm weight compensation over the workspace.

## Methods

### Arm weight compensation methods

#### Average arm method (Average)

**Summary** - This method models the arm as two connected rigid segments, corresponding to upper and lower arm, with no additional torques/forces at the joints. The human arm tendon forces are neglected. The parameters of these two segments, weight and center of mass (CoM) location, are adjusted manually with the information from anthropometric tables for each subject. Therefore, the therapist enters their height/weight information in the PC interface for calibration of the method. As a simplification, the locations of CoMs are assumed to lie on the rotational axes of the arms. Once these weight and CoM parameters are adjusted, the robot compensates the arm weight by applying appropriate torques at each pose, using the varying pose information of the robot (therefore the human arm), basic geometry, and mathematical calculations.

**Details** - The upper arm (*ua*) and lower arm (*la*) are treated as two independent, rigid segments that are connected to the robot. The weight of each arm segment is compensated with the torques obtained from a model using the anthropometric table approximation values: mass $\tilde {m}$ and center of mass ($\tilde {CoM}$) location vector according to the cuff location, ${}^{c}\tilde {\boldsymbol {r}}$. A wrench vector ${\tilde {\boldsymbol {\omega }}_{weight}}$ is then constructed at each cuff frame *c* at upper arm, (*c*=*c*_*ua*_), and lower arm, (*c*=*c*_*la*_), as follows:

1 $$  {}^{c}\tilde{\boldsymbol{\omega}}_{weight} = \left[\begin{array}{c} \boldsymbol{R}_{0}^{c} \textbf{} {}^{0}\tilde{\boldsymbol{f}}\\[0.3em] {}^{c}\tilde{\boldsymbol{r}} \times \boldsymbol{R}_{0}^{c} \textbf{} {}^{0}\tilde{\boldsymbol{f}} \end{array}\right],  $$

where ${}^{0}\tilde {\boldsymbol {f}}= \left [ 0,0, \tilde {f} = -\tilde {m}g \right ]^{T}$ is the approximated weight vector, including the gravitational constant *g*, in the earth frame (*x*_0_,*y*_0_,*z*_0_) and $\boldsymbol {R}_{0}^{c}$ is the rotation matrix rotating from the earth frame to the respective cuff frame. The wrench vector $\tilde {\boldsymbol {\omega }}_{weight}$ of the upper and lower arm is mapped to the joint space of the robot by multiplication with the respective Jacobian at the cuff location at the upper arm, ${}^{c_{ua}}\boldsymbol {J}^{T}_{c_{ua}}$, and at the lower arm ${}^{c_{la}}\boldsymbol {J}^{T}_{c_{la}}$ to obtain the arm weight compensation torque ***τ***_*comp*_:

2 $$ \begin{aligned}  {\boldsymbol{\tau}}_{\text{comp}}= &-{\!~\!}^{c_{ua}}\boldsymbol{J}^{T}_{{c}_{\text{ua}}} {\!~\!}^{{c}_{\text{ua}}}\tilde{\boldsymbol{\omega}}_{\text{weight,ua}} \\ &-{\!~\!}^{{c}_{\text{la}}}\boldsymbol{J}^{T}_{{c}_{la}} {\!~\!}^{{c}_{la}}\tilde{\boldsymbol{\omega}}_{\text{weight,la}}. \end{aligned}  $$

**Fig. 1:**
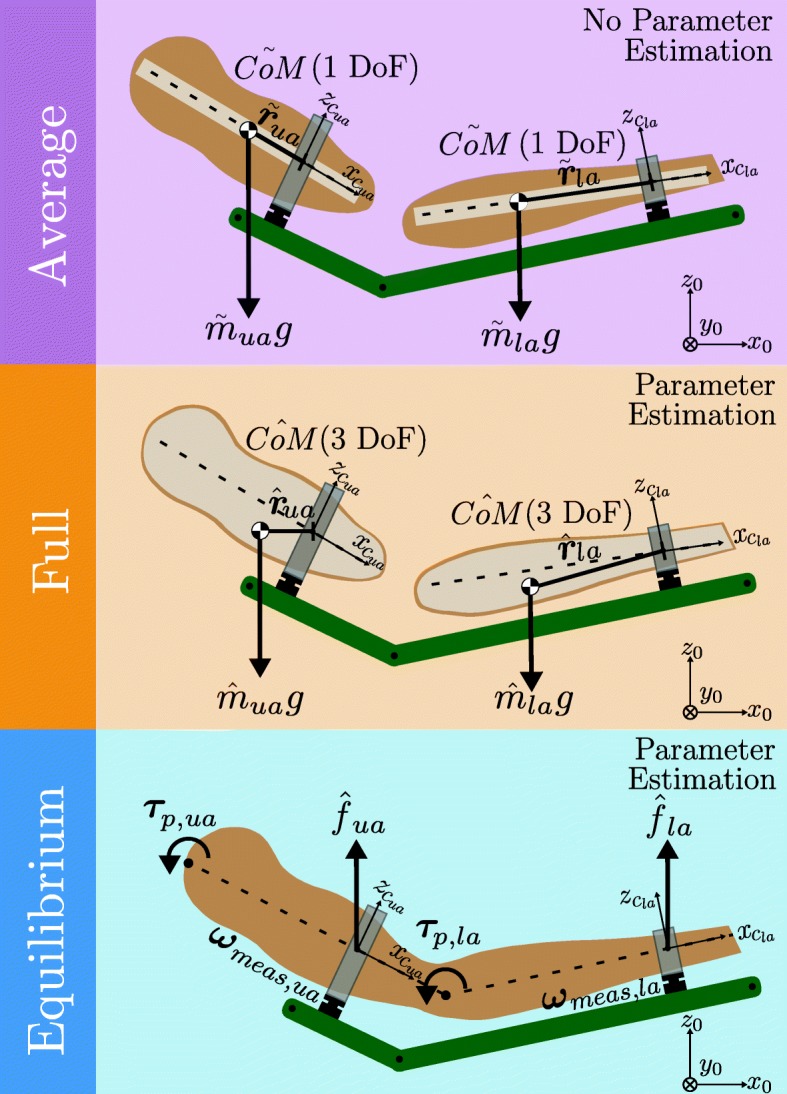
The three arm weight compensation methods: Method *Average*: Masses $\tilde {m}$ and center of masses ($\tilde {CoM}$) are approximated through anthropometric tables based on basic information of the subject. The arm is modelled as two independent rigid bodies, upper arm (*ua*) and lower arm (*la*). The *CoMs* of the objects are assumed to lie on the arm segment rotation axis. Method *Full*: The parameters $\hat {m}$ and $\hat {CoM}$ are estimated through force/torque sensor data at each cuff. The arm is modeled as two independent rigid bodies, similarly to method *Average*, though the $\hat {CoM}s$ of each object are not assumed to lie on arm segment rotation axis and are fully estimated. Method *Equilibrium*: A quasistatic equilibrium against gravity is created at each cuff. Upper and lower arm are assumed to be connected to each other. The passive joint torques ***τ***_*p*,*ua*_ and ***τ***_*p*,*la*_ are partially taken into account. The support force parameters $\hat {f}_{ua}$ and $\hat {f}_{la}$ are calculated and achieve an equilibrium against the wrenches created by gravity at the cuffs, *ω*_*meas*,*ua*_,*ω*_*meas*,*la*_. The *CoMs* of the arm are assumed to lie on each arm segment rotation axis

The calculated arm parameters (masses $\tilde {m}_{ua}$, $\tilde {m}_{la}$ and center of mass (${\tilde {CoM}}$) location vectors $\boldsymbol {\tilde {r}}_{ua}$, $\boldsymbol {\tilde {r}}_{la}$) in the method *Average* are taken directly from anthropometric tables and are not estimated [34, 35] (see Fig. 1). The anthropometric tables provide an approximation of the upper and lower arm mass as well as the location of the *CoM* based on the subject’s height and weight. The therapists must manually enter height and weight information for each new subject to calculate the arm parameters. Therefore, neither an estimation procedure, nor force/torque sensors are needed. The method can be applied to any rehabilitation device that allows for compensation of arm weight by active mechanisms.

There are no external forces applied on these rigid objects. In other words, the passive torques at the joints created by the surrounding tissues, muscles, tendons, etc., are assumed to be zero. Furthermore, the *CoMs* of both the upper and lower arms are assumed to be on the axes of internal/external shoulder rotation and forearm pronation/supination, respectively. Thus, only one dimension ($x_{c_{ua}}$, $x_{c_{la}}$) of the three dimensional *CoM* vectors for both the upper and lower arm is adapted, while the other two dimensions are always assumed to be zero, ${}^{c}\hat {\boldsymbol {r}}= {}^{c}\left [\hat {r}_{x}, 0, 0 \right ]^{T}$ (see Fig. 1). For a more detailed explanation, mathematical derivation and implementation, interested readers are referred to the Additional file 1 and our previous work [13].

#### Full estimation method (Full)

**Summary** - This method is similar to the *Average* method with two main differences; the CoM locations of the arm segments are not assumed to lay on the rotational axes of the arms. Therefore, CoM locations are vectors with three elements each, rather than one. Secondly, the arm parameters are not adjusted with anthropometric table information; they are estimated for each subject using force sensor data. During a calibration phase, the robot maintains two predefined poses, while the subject keeps his/her arm slack for 10 s inside the robot. This method estimates subject-specific arm parameters to increase the efficacy of the arm weight compensation.

**Details** - This method is based on a model that assumes that the arm is composed of the two independent segments, an upper arm and a lower arm, which are rigidly connected to the robot. The weight vector ${}^{0}\hat {\boldsymbol {f}}= \left [ 0,0, \hat {f} = -\hat {m}g \right ]^{T}$ and the location of the $\hat {CoM}$ relative to the cuff location, ${}^{c}\hat {\boldsymbol {r}}= {}^{c}\left [\hat {r}_{x}, \hat {r}_{y}, \hat {r}_{z} \right ]^{T}$, are estimated through the force/torque sensor data and are not approximated as in (1) and (2):

3 $$  {~}^{c}\hat{\boldsymbol{\omega}}_{\text{weight}} = \left[\begin{array}{c} \boldsymbol{R}_{0}^{c} {~}^{0}\hat{\boldsymbol{f}}\\[0.3em] {~}^{c}\hat{\boldsymbol{r}} \times \boldsymbol{R}_{0}^{c} {~}^{0}\hat{\boldsymbol{f}} \end{array}\right],  $$

4 $$ \begin{aligned}  {\boldsymbol{\tau}}_{\text{comp}}= &-{~}^{{c}_{\text{ua}}}\boldsymbol{J}^{T}_{{c}_{\text{ua}}} {~}^{{c}_{\text{ua}}}\hat{\boldsymbol{\omega}}_{\text{weight,ua}} \\ &-{~}^{{c}_{\text{la}}}\boldsymbol{J}^{T}_{{c}_{\text{la}}} {~}^{{c}_{\text{la}}}\hat{\boldsymbol{\omega}}_{\text{weight,la}}. \end{aligned}  $$

All the estimated parameters of the method ($\hat {m}$, $\hat {r}_{x}$, $\hat {r}_{y}$, $\hat {r}_{z}$) are assumed to be nonzero (see Fig. 1). The arm parameters must be estimated once at the beginning of therapy in two static poses for calibration using force/torque sensor data. During this process, it is crucial that the subject does not apply any force to ensure accurate estimation results of the arm weight compensation method. The robot moves the passive arm to two arm poses by position control, while the arm parameters are estimated; for more details, please refer to the Additional file 1.

#### Equilibrium estimation method (Equilibrium)

**Summary** - This method combines the model from the *Average* method and uses force sensor data to estimate the model parameters like the *Full* method. During a calibration phase, the robot maintains a single predefined pose, while the human keeps his/her arm slack for 10 s in the robot. However, instead of estimating the mass *m* and center of mass (CoM) location parameters separately, it focuses on the total effect of the arm weight on the robot joints. The method estimates fixed support forces at the cuff locations that cancel the human arm weight and create a force/torque equilibrium for the arm. This method estimates only one parameter each for upper and lower arm to minimize possible estimation errors, while still partially compensating for the forces/torques at the joints (e.g. tendon forces, muscle tone).

**Details** - For the compensation of the weight of the arm, ***τ***_*comp*_, the method *Equilibrium* applies two estimated virtual support wrenches, each consisting of a single force parameter, at the sensor locations on the upper and lower arm ($\hat {f}_{ua}$, $\hat {f}_{la}$) (see Fig. 1). These support wrenches support the arm against gravity (*z*_0_ direction):

5 $$  {\boldsymbol{\tau}}_{\text{comp}}= {~}^{0}\boldsymbol{J}^{T}_{c_{\text{ua}}} \left[\begin{array}{c} 0\\ 0\\ \hat{f}_{\text{ua}} \\ 0\\ 0\\ 0\\ \end{array}\right] + {~}^{0}\boldsymbol{J}^{T}_{c_{\text{la}}} \left[\begin{array}{c} 0\\ 0\\ \hat{f}_{\text{la}}\\ 0\\ 0\\ 0\\ \end{array}\right],  $$

where ${}^{0}\boldsymbol {J}^{T}_{c_{ua}}$ and ${}^{0}\boldsymbol {J}^{T}_{c_{la}}$ are the Jacobians in the earth coordinate frame that project the support wrenches on the robot joints.

The *Equilibrium* method estimates the values for the two force parameters $\hat {f}_{ua}$ and $\hat {f}_{la}$ of the two wrenches while the subject’s arm is passive and fixed by the robot in a single pose. This calibration procedure is performed once in the beginning of the therapy. A torque equilibrium is formed using the measured interaction joint torques:

6 $$ \boldsymbol{\tau}_{\text{meas}}= {~}^{0}\boldsymbol{J}^{T}_{{c}_{\text{ua}}} {~}^{0}\boldsymbol{\omega}_{\text{meas,ua}} + {~}^{0}\boldsymbol{J}^{T}_{{c}_{\text{la}}} {~}^{0}\boldsymbol{\omega}_{\text{meas,la}}.  $$

The measured interaction wrenches at the physical human-robot interaction points at upper, ^0^***ω***_*meas*,*ua*_, and lower arm, ^0^***ω***_*meas*,*la*_, are multiplicated with the respective Jacobian in the earth coordinate frame to project torque on the robot joints. The torque equilibrium of the interaction joint torques ***τ***_*meas*_ is used to estimate the arm weight-related torques on the robot joints caused by the force parameters.

7 $$  \boldsymbol{\tau}_{\text{meas}}+{~}^{0}\boldsymbol{J}^{T}_{{c}_{\text{ua}}} \left[\begin{array}{c} 0\\ 0\\ \hat{f}_{ua} \\ 0\\ 0\\ 0\\ \end{array}\right] + {~}^{0}\boldsymbol{J}^{T}_{{c}_{\text{la}}} \left[\begin{array}{c} 0\\ 0\\ \hat{f}_{\text{la}}\\ 0\\ 0\\ 0\\ \end{array}\right] =0.  $$

The method is built on the following assumptions: The two arm segments cannot be considered as two separated segments. Accordingly, passive joint torques between body segments cannot be neglected, even in a passive and relaxed arm pose [36]. Passive joint torques are not assumed to be zero in the *Equilibrium* method. The upper arm is connected to the shoulder, and the lower arm is connected to the upper arm, as in real world scenarios. The passive torques the shoulder applies on the upper arm, ***τ***_*p*,*ua*_, and the passive torques the upper arm applies on the lower arm, ***τ***_*p*,*la*_, are nonzero and partially included through the torque ***τ***_*meas*_ measured during the calibration procedure (see. (7)). For the *Equilibrium* method, the passive joint torques are assumed to point around the global *y*_0_ axis direction in all configurations and the $\hat {CoMs}$ of the arm are assumed to lie on the rotation axes (the same as in the *Average* method). Therefore, only the joint torques that are in the gravity direction are considered and partially compensated. The joint torques contain tendon forces, torques due to muscle tone, and other influences. As a future research step, these elements could be modeled to improve the model.

The torque equilibrium in (7) is used to estimate the support force parameters. Upon careful examination, one will notice that there are more equations resulting from this equilibrium equation than number of variables (two). In other words the equation system is over-determined. Therefore, in order to determine the parameters, weighted least squared method is used. The weights of the equations are selected according to the magnitudes of the Jacobian values of each equation in (7) such that if the Jacobian values are close to zero, the corresponding weight is also close to zero. This way, estimation errors due to sensor noise are minimized. Once these force parameters are determined, they are multiplied with the Jacobians of the respective cuff and applied to the motors, ***τ***_*comp*_, to compensate for the patient’s arm weight in each pose (see (5)).

#### A conceptual overview of the three methods

An conceptual overview of the differences between the methods is presented in Table 1.

**Table 1:** Overview and conceptual comparison of all three weight compensation methods: If an estimation is performed, the number of estimation poses needed the calculated degree of freedom of the center of mass, the usage of force/torque sensors, the costs of needed hardware, the number of parameters needed for each method, whether passive joint torques are considered, and the relative effort of calibration

	*Average*	*Full*	*Equilibrium*
Estimation	No	Yes	Yes
*#*of estimation poses	No	2	1
*CoM* DoF calculation	1	3	0
Force/torque sensors	No	Yes	Yes
Hardware costs	Low	High	High
Number of parameters	4	8	2
Passive joint torques	No	No	Partial
Calibration effort	Low	High	Medium

The differences in hardware costs for all three methods are purely based on the non/-usage of force/torque sensors. The prices of currently available standard sensors are high. Apart from the extra hardware costs, integrating estimation of patient-specific parameters requires extra calibration (estimation) time in exchange for possibly higher efficacy for the methods. The calibration procedure of the *Average* method needs the therapist to enter patient’s height and weight information in the PC, the calibration procedure of the *Equilibrium* and *Full* methods need 10 s of patient’s arm weight data passively held by the robot in 1 or 2 poses, respectively. Varying and high muscle activity of the arm during the passive calibration of arm weight is a possible patient error during calibration of the *Full* and *Equilibrium* methods. This would lead to higher estimation errors and, therefore, lead to a necessary repetition of the calibration procedure. The calibration time and effort of method *Average* is the lowest, because it doesn’t need recorded arm weight data and it is always reproducible. The parameter estimation of methods *Full* and *Equilibrium* are reproducible within limits that are discussed in more detail in a sensitivity analysis.

### ARMin rehabilitation robot

The ARMin IV+ exoskeleton robot has seven actuated DoFs [37]: *θ*_1_ (horizontal shoulder abduction/adduction), *θ*_2_ (shoulder elevation), *θ*_3_ (internal /external shoulder rotation), *θ*_4_ (elbow flexion/extension), *θ*_5_ (forearm pronation/supination), *θ*_6_ (wrist flexion/extension), and *θ*_7_ (hand opening/closing). In this paper, the first five axes are used and the hand module containing axis 6 (*θ*_6_) and 7 (*θ*_7_) are removed. Six DoF force/torque sensors are attached at the two physical human-robot interaction points at the upper and lower arm (F/T Sensor: Mini45, ATI Industrial Automation, Apex, USA). To achieve a high level of underlying robot transparency for physical human-robot interactions, a velocity-based disturbance observer was implemented, which has been shown to achieve high transparency with low intersubject variability and reliable performance across different movement velocities [23].

### Ethics

The following three studies were approved by the responsible institutions (KEK-ZH-Nr. 2015-0013, Zurich, Switzerland; clinicaltrials.gov NCT02720341).

## Sensitivity analysis of the parameter estimation

### Study design: sensitivity analysis of the parameter estimation

The *Full* and *Equilibrium* methods are both based on force/torque sensor data, and therefore, the arm weight and center of mass need to be estimated with the subject inside the ARMin robot. Since the estimation quality differs for different subject anthropometries and different estimation poses, an analysis of the estimation behavior to achieve high and robust estimation quality is needed. First, the temporal changes in the estimation results, called temporal sensitivity, were evaluated by the standard deviation of the estimation results over time. Second, the influence of the estimation pose on the estimation result, called spatial sensitivity, was tested.

For evaluation of the spatial and temporal sensitivities of the *Full* and *Equilibrium* methods, the subject’s passive arm was driven by the position-controlled ARMin in *p*=27 different poses. The 27 poses were defined to cover the functional arm range of motion (RoM) of healthy subjects [38]. Each pose is measured only once per subject, since the physical human-robot interface cuffs during ARMin usage are always attached tightly to the human to prevent slipping during movement. In each of these poses, the weight of the arm was measured over six seconds during each iteration with the 1800 Hz real-time system. Subsequently, the arm weight data were used to calculate the estimation results of each method. Since one estimation pose is needed for the *Equilibrium* method, the *p*=27 poses were evaluated in a straightforward manner. The *Full* method requires a pair of poses. Therefore, estimation results of all possible pose-pairs (2-permutations of 27 as binominal coefficients), $\left (\begin {array}{c}27\\2\end {array}\right)= \frac {27!}{2!(27-2)!}=351$are calculated after making the 27 pose measurements. For the *Equilibrium* and *Full* methods all pose-pairs and poses are analyzed for temporal and spatial sensitivity, respectively.

### Primary outcome: sensitivity analysis of the parameter estimation

The temporal sensitivity analysis is focused on the standard deviation of the estimated arm weight compensation forces $\left (\hat {f}_{ua}, \hat {f}_{la} \right)$ and their estimated centers of mass location vectors ($\boldsymbol {\hat {r}}_{ua}$, $\boldsymbol {\hat {r}}_{la}$) over time. Thus, imprecise estimation poses with estimations comprising high standard deviations outside possible biological arm values should be identified.

The spatial sensitivity analysis focused on the average standard deviation of the estimated parameters for all subjects over all *p*=27 arm poses. Furthermore, an algorithm identified the estimation pose (for the *Equilibrium* method) or pose-pair (for the *Full* method) that minimized the performance variability over the workspace since the subject’s real arm weight is not known. The data acquisition (6*s* measurement, 10*s* pose to pose movement) of all 27 calibration poses takes 7 min (27·16*s*) of therapy time, therefore, as a fast calibration solution in therapy the methods should just depend on the best estimation pose or the best pose-pair, found respectively. The goals of the algorithm are that the estimated force parameters are within the threshold of *δ*_*f*_=4*N* with respect to the directly measured force signal of the force/torque sensor in the direction of gravity (*f*_*ua*_,*f*_*la*_) and that they closely match the overall mean of all arm weight estimation results. This way also singular estimation poses are excluded that would bias the mean of all estimation results. For more details including the joint angles of all tested poses, please refer to the Additional file 2.

### Subjects: sensitivity analysis of the parameter estimation

The sensitivity analysis was performed with 5 healthy subjects (3 females; 5 right-handed; age: 24.50 ±2.17 years; height: 177.50 ±10.21 *m*; weight: 69.67 ±10.71 *kg*). Their left and right arm were considered for the evaluation, resulting in a total of 10 arm data sets. The inclusion criteria were at least 18 years of age, no serious medical or psychiatric disorders, no cybersickness, no pacemaker or other implanted electric devices, and a body weight less than 120 *kg*.

### Results: sensitivity analysis of the parameter estimation

The temporal sensitivity analysis for the *Full* and *Equilibrium* methods revealed the following mean standard deviation of the estimated force parameters for upper and lower arm ($\textit {Full:} \mu _{\sigma _{\hat {f}_{ua}}} =0.34 N, \mu _{\sigma _{\hat {f}_{la}}}=0.13 N, \textit {Equilibrium:} \mu _{\sigma _{\hat {f}_{ua}}}=0.54 N, \mu _{\sigma _{\hat {f}_{la}}}=0.19 N)$. The mean standard deviation of the estimated force parameters was below 3% of an average arm weight (height:175 cm, weight: 70 kg, $\tilde {f}_{ua}=20N, \tilde {f}_{la}=15N,$ [35]) for the upper and lower arm. Additionally, several estimation pose-pairs of the *Full* method could be identified with standard deviations for the *CoM* of the upper arm bigger than several meters. More details are shown in the Additional file 2.

**Fig. 2:**
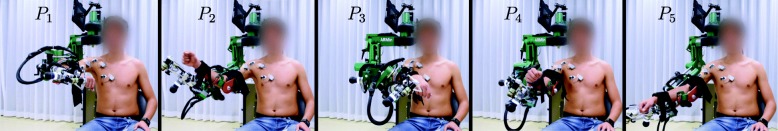
The five EMG measurement poses (*P*_1_,...,*P*_5_) for the efficacy comparison study: The poses represent the ADL usage of a stroke subject: *P*_1_) Mouth and head reaching (-45,90,90,100,0), *P*_2_) Ipsilateral reaching on shoulder level (-45,70,5,60,0), *P*_3_) Contralateral reaching (10,70,30,40,60), *P*_4_) Medial reaching at shoulder level (-15,65,15,50,0), *P*_5_) Ipsilateral reaching on abdomen level (-50,55,30,40,0). Pose coordinates of the axes (1,2,3,4,5) in degree are according to anatomical axes definitions [39]. Axis 1 (horizontal shoulder abduction/adduction), axis 2 (shoulder elevation), axis 3 (internal /external shoulder rotation), axis 4 (elbow flexion/extension), and axis 5 (forearm pronation/supination). *P*_1_ and *P*_2_ are the chosen estimation poses (14,20) of the sensitivity analysis and *P*_3_, *P*_4_ and *P*_5_ complement the workspace for stroke patients in ARMin. All coordinates of the poses of the sensitivity analysis and efficacy comparison study are shown in the Additional file 2

The spatial sensitivity analysis for the *Full* and *Equilibrium* methods revealed a mean standard deviation of the arm estimation forces below 23% of the same average arm weight for the upper and lower arm ($\textit {Full}:\mu _{\sigma _{\hat {f}_{ua}}}=3.89 N, \mu _{\sigma _{\hat {f}_{la}}}=2.35 N, \textit {Equilibrium}: \mu _{\sigma _{\hat {f}_{ua}}}=6.74 N, \mu _{\sigma _{\hat {f}_{la}}}=3.55 N$). The used misalignment avoidance algorithm revealed that the pose-pair 14 and 20 (*P*_1_ and *P*_2_) yielded the best algorithm results for the *Full* method, while pose 14 (*P*_1_) yielded the best estimation pose for the *Equilibrium* method (see Fig. 2). The *Full* and *Equilibrium* methods used this respective best pose/pose-pair to estimate arm weight for the following arm weight compensation methods efficacy comparison in healthy subjects.

### Discussion: sensitivity analysis of the parameter estimation

According to the temporal sensitivity analysis, the magnitude of the standard deviation of the estimation results over time corresponds to 3% of an average human arm weight. This is a precise estimation behavior. Following, the variation in the passive joint torques and human reflexes in arbitrary poses over time are negligible for the arm weight force parameter estimations in healthy subjects. However, the *Full* method showed high variance for the estimated upper arm *CoM* location vectors ***r*** that were mainly caused by several pose-pairs leading to nonconvergence of the method’s algorithm and to infeasible results. The nonconvergence is probably due to the similarity of both pose-pair poses, leading to singularities in the estimation calculations. All poses and singular pose-pairs are presented in the Additional file 2.

The spatial sensitivity analysis of both methods revealed pose-dependent estimation differences with around 23% mean of the standard deviation of the estimation results projected on an exemplary average arm weight. This is probably due to the simplifications of the arm weight compensation models for the *Full* and *Equilibrium* methods. Therefore, the developed misalignment avoidance algorithm presented in the Additional file 2 found the pose and pose-pair with least disturbing torques that was taken as estimation pose-pair and pose for the *Full* and *Equilibrium* methods. For the following studies the mean is taken over six seconds in the robot-controlled estimation pose-pair/pose for the model estimation parameters. The arm muscles need to be passive and the arm is passively hold in the calibration pose by the position-controlled robot. Please note it is crucial for the subject inside to stay more or less passive during the data acquisition; at least not actively moving. Otherwise it is not possible to decouple the weight of the arm from other forces the subjects apply. The result of this calibration procedure would probably be similar to the results obtained by using an adaptive controller to estimate the weight. However, similarly, care has to be taken to make sure that the subject stays passive otherwise the arm weight is not extractable from the force sensor data.

## Efficacy comparison of the arm weight compensation methods

### Study design: efficacy comparison of the arm weight compensation methods

The efficacies of the three presented methods in effectively relieving each subject’s arm weight for arm weight compensation inside the ARMin robot were evaluated. Approximately 15 s of EMG signals were acquired from six upper limb muscles that are important for maintaining the arm pose against gravity during each condition and pose. Each subject was instructed to hold his/her arm in five poses that covered the workspace of ARMin.

Data were acquired in each pose in randomized order in five different conditions. In the first condition, the robot was position-controlled and the subject fully relaxed his/her arm in the exoskeleton ARMin (passive condition). In the second condition, the subject had to hold his/her arm against gravity while ARMin operated in transparent control mode (active condition) [23]. The subject was told to maintain the pose with minimal effort. The three following, randomized measurements were made with the three arm weight compensation methods previously described and the subjects were again told to maintain their pose with minimal effort. Summarized for each randomized pose, a passive and an active condition were tested subsequently. Then, the three arm weight compensation methods were tested in a random order.

Two of the five tested poses were the estimation poses found as a result of the sensitivity analysis for the *Full* method and the *Equilibrium* method. The three other poses should complement the functional RoM of stroke patients (see Fig. 2). Furthermore, they should represent some of the most important reaching poses also performed in activities of daily living (ADLs). The robot automatically guided the arm via position-control to the next pose between the measurements. Then, a position-controlled resting time of 10 s was provided before the next measurement was started. The subjects received auditory feedback, when each measurement was started. Each condition was measured once per subject.

### EMG signal processing: efficacy comparison of the arm weight compensation methods

Surface EMG signals are recorded with a commercial 16-channel electromyographic system, Telemyo DTS (Noraxon, USA) and the sampling frequency was set to 1500 Hz. The EMG system uses EMG preamplifiers with a gain of 500, the common mode rejection ratio (CMRR) is higher than 100 dB, and the input impedance higher than 100 M*Ω*. Dual EMG surface self-adhesive Ag/AgCl electrodes (Noraxon, USA) were placed at a constant inter-electrode distance of 1.75 cm on the belly of six upper-limb muscles, along the longitudinal direction as shown in Fig. 2. Recordings were acquired from the pectoralis major (PM, shoulder horizontal flexor), the upper trapezius (UT, shoulder elevator), the posterior deltoid (PD, shoulder extensor), the anterior deltoid (AD, shoulder flexor), the biceps brachii (BB, elbow flexor) and the lateral triceps (LT, elbow extensor) as reported in [13].

### Primary outcomes: efficacy comparison of the arm weight compensation methods

To evaluate the efficacy of the arm weight compensation methods, each condition was tested in each pose with EMG activity recordings one time for 15 s. Each EMG recording of 15 s was shortened by 1/3 (1/6 of the samples at the beginning and end were removed) to guarantee stationary data.

For the remaining 10 s of data, the smooth rectified EMG (SRE) using linear envelopes was calculated. For further analysis the mean of the SRE ($\overline {\text {SRE}}$) was taken. In each pose *j* and for each muscle *i*, the mean SRE ($\overline {\text {SRE}}$) values of a passive condition with the position-controlled robot as a lower limit ($\overline {\text {SRE}}_{\text {passive},i,j}$) and of an active, isometric test condition with a transparent robot as upper limit ($\overline {\text {SRE}}_{\text {active},i,j}$) were used to normalize the $\overline {\text {SRE}}_{i,j}$ values of each method to between *"*0*"* and *"*1*"*, called $\overline {\text {nSRE}}_{i,j}$:

8 $$ \overline{\text{nSRE}}_{i,j} = \frac{\overline{\text{SRE}}_{i,j} - \overline{\text{SRE}}_{\text{passive},i,j}}{\overline{\text{SRE}}_{\text{active},i,j} - \overline{\text{SRE}}_{\text{passive},i,j}}.  $$

For computation of the overall effort of the subject in each pose *j*, the mean effort index (MEI) [13]

9 $$ \text{MEI}_{j}=\frac{1}{\sum_{i=1}^{6} w_{i,j}}\sum_{i=1}^{6} w_{i,j} \overline{\text{nSRE}}_{i,j}, \;\; \forall j \in \left[ 1,...,5 \right]  $$

was computed for each pose *j* as a weighted average of the $\overline {\text {nSRE}}$-values of the 6 tested muscles. The weights were computed as follows, so that the muscles that were more physiologically involved in the weight support have more influence in the effort estimation

10 $$ w_{i,j}=1-\frac{\overline{\text{SRE}}_{\text{passive},i,j}}{\overline{\text{SRE}}_{\text{active},i,j}} \,, \forall i \in \left[ 1,...,6 \right], \forall j \in \left[ 1,...,5 \right].  $$

**Fig. 3:**
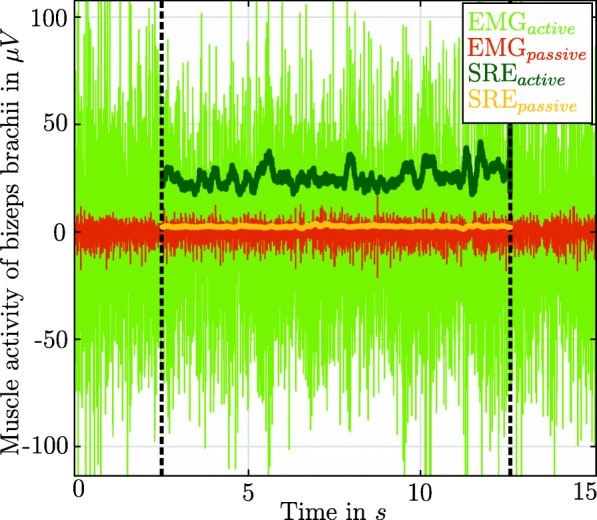
Data interpretation of raw EMG data to the smooth rectified EMG (SRE): Raw EMG data of a bizeps brachii measurement in position *P*_1_ for the active and passive reference measurement (SRE_active_, SRE_passive_). The dashed lines correspond to the shortening of EMG recording 2.5 s in the beginning and end. Finally, the SRE is calculated as the linear envelope of the selected raw EMG data

The MEI represents the subject’s mean effort: Accordingly, the lower the MEI, the less force is required for the patient to maintain his/her arm in the particular pose. Therefore, the primary outcome measure assesses the efficacy in removing the subject’s arm weight including method induced disturbances (Fig. 3).

### Subjects: efficacy comparison of the arm weight compensation methods

The study was performed with 31 healthy subjects (9 females; 29 right-handed; age: 25.09 ±2.28 years; height: 177.28 ±8.95 *cm*; weight: 70.81 ±11.23 *kg*). The dominant arm was evaluated in the study. Inclusion criteria were at least 18 years of age, no serious medical or psychiatric disorders, no cybersickness, no pacemaker or other implanted electric devices, and body weight less than 120 kg.

### Statistical methods: efficacy comparison of the arm weight compensation methods

The statistical analysis was performed with R (version 3.4.2, R Core Team, 2017). Linear mixed effect models were used with “lme4” [40] to assess the relationship between EMG activity and the different pose- and method combinations. As each subject has different anatomical and physiological features which can influence the signal detected by the surface electrodes, a random intercept for the effect of the different subjects was introduced into the statistical model. Chi-squared-based p-values were obtained with ANOVA to compare all of the statistical models for a given possible effect. The resulting mixed effect statistical model includes *method* and *pose* effects:

11 $$ \text{MEI} \sim method + pose + (1 | subject).   $$

The statistical model assumptions were checked through visual examination of residuals plots by experts. Additionally, a Dunnet’s test for multiple comparisons was carried out using “multcomp” [41] to test for possible significant differences between methods. Holm’s method was applied to adjust for multiple comparisons. The statistical significance level was set to *p*<0.05 (’*’). A p-value of *p*<0.001 (’***’) corresponds to a highly significant result.

### Results: efficacy comparison of the arm weight compensation methods

**Fig. 4:**
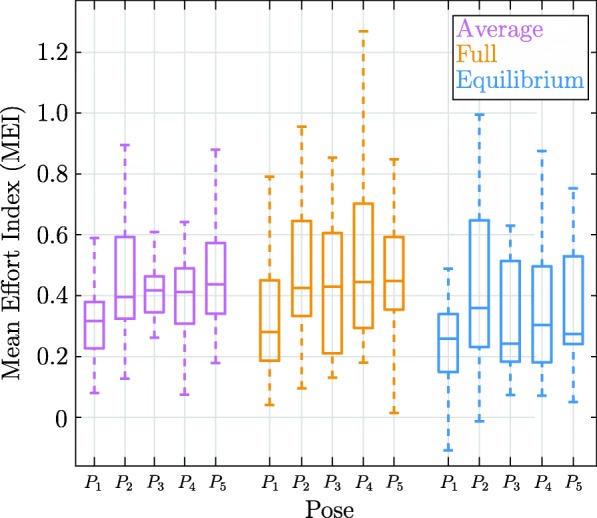
Mean effort index (MEI) results of the methods for all poses over all subjects: The boxplot shows the subjects’ activity according to the MEI over the five tested poses *P*_*i*_ for each of the three arm weight compensation methods in healthy subjects. The whiskers end at the most extreme data points, the median is displayed, and the bottom and top box edges show the 25th and 75th percentile respectively

**Fig. 5:**
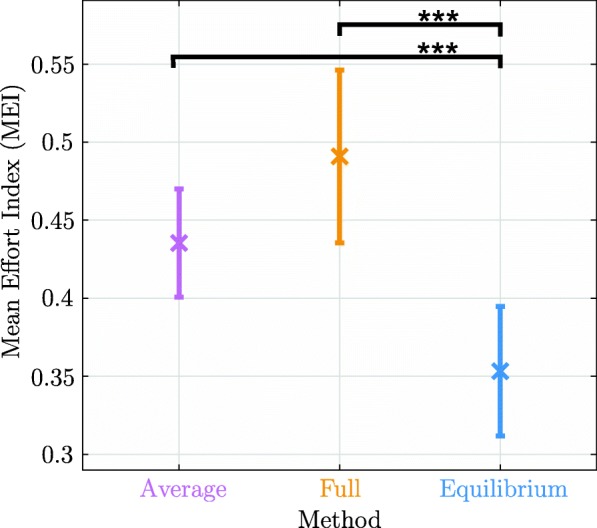
Confidence intervals of the Mean effort index (MEI) results of the methods over all subjects and poses: The plot shows the MEI mean in 95*%* confidence intervals for the three arm weight compensation methods summarized for all participants and poses. Method *Average*: 0.435±0.035 MEI, method *Full*: 0.491±0.055 MEI, and method *Equilibrium*: 0.353±0.041 MEI. Dunnet’s multiple comparison revealed that method *Equilibrium* is highly significantly better as method *Average* (*p*<0.001) and method *Full* (*p*<0.001) respectively

As shown in Fig. 4, an increase in the median MEI from the *Average* method to the *Full* method was present, except for pose *P*_1_. Additionally, *P*_1_ possessed the overall lowest median MEI values for every method. The *Equilibrium* method had the lowest MEI median for all of the five tested poses. For position *P*_1_, the *Equilibrium* method had the lowest of all MEI medians and the lowest upper and lower quartiles and whiskers. The confidence interval plot in Fig. 5 shows that the *Average* method had the lowest standard deviation (0.035 MEI) compared to the *Average* method (0.055 MEI) and the *Average* method (0.041 MEI). The *Full* method had the highest MEI mean and the *Equilibrium* method had the lowest MEI mean. The upper confidence interval bound of the *Equilibrium* method was below the lower confidence intervals of the *Full* method and the *Average* method.

The Dunnet’s test revealed that the *Equilibrium* method highly significantly reduced the mean EMG activity compared to the *Average* method (*p*<0.001) and the *Full* method (*p*<0.001). More precisely, differences of 0.082 MEI (95% confidence interval 0.027-0.137) between the *Equilibrium* method and the *Average* method and of 0.138 MEI (95% confidence interval 0.083-0.192) between the *Equilibrium* method and the *Full* method were identified. The statistical model presented in (11) revealed no significant interaction between *method* and *pose*. Table 2 summarizes the arm weight compensation method characteristics and the main results of the arm weight compensation comparison in healthy subjects.

**Table 2:** An overview of the study results and details of all three weight compensation methods

	Average	Full	*Equilibrium*
MEI reduction	Second	Third	Best
Lowest variability	Best	Third	Second
Estimation	No	Yes	Yes
Calibration time	Low	Medium	Low
Number of parameters	4	8	2
Hardware costs	Low	High	High

### Discussion: efficacy comparison of the arm weight compensation methods

The differences in MEI for each pose show that all three algorithms perform differently in the evaluated workspace (see. Fig. 4). Since each subject has different anatomical and physiological predispositions that influence the signals detected by the surface electrodes, a higher intersubject variability can be observed in Fig. 4. However, no significant interaction was found between weight compensation method and pose, i.e., no simple systematic effect could be shown. That means that other factors such as passive joint torques (e.g., shoulder stiffness), and other disturbing factors (e.g., pose-dependent twisting of the upper and lower arm in the cuffs of the robot) could be responsible for the changing muscle activity in different poses and should be considered in further research.

The *Average* method showed the lowest median MEI value in pose *P*_1_ (see Fig. 4). This result is consistent with the result of the sensitivity analysis, since *P*_1_ is the pose with the least disturbing forces and furthermore, indicates the validity of the spatial sensitivity algorithm. Figures 4 and 5 show that the *Average* method has the lowest intersubject variability, leading to more steady performance of the algorithm for each subject than the two force/torque sensor based methods. The errors introduced by the anthropometric tables of the *Average* method seem to have a smaller influence on the MEI variance than estimation errors of the other two estimation methods, since the *Average* method confidence interval in Fig. 5 is the smallest.

The *Full* method also showed its lowest median MEI value in pose *P*_1_ (see Fig. 4). Since poses *P*_1_ and *P*_2_ are the arm weight compensation estimation poses, it is expected that these poses will yield better results in the study than the complementary poses. However, the median MEI of estimation pose *P*_2_ and the three remaining complementary poses are on similar levels, suggesting that the *Full* method was successful in the whole workspace outside of the calibration poses. Furthermore, the *Full* method shows the highest variance in Fig. 5, probably because the algorithm that requires two different estimation poses and the additional calculation of two full *CoM* vectors, resulting in higher variance. Overall, the passive joint torques and the other disturbing torques, such as twisting of the upper and lower arm in the cuff, seem to influence the more detailed and combined estimation of force parameters and *CoM* vectors of the *Full* method more intensively, leading to lower estimation quality and resulting in lower performance.

The *Equilibrium* method has the lowest mean MEI and the second lowest variance (see Fig. 5). Therefore, subjects using the *Equilibrium* method for arm weight compensation can expect the highest performance with low intersubject variability in every pose. Figure 4 reveals that *P*_3_ has the lowest median MEI, however, the estimation pose *P*_1_ would be expected to have the best performance and the lowest median MEI. Overall, the similar MEI distribution over all poses underlines that the method is valid outside of the estimation pose *P*_1_ and shows successful results over the tested workspace of ARMin (see Fig. 4). Dunnet’s multiple comparison in Fig. 5 using the derived linear mixed effect model showed that the *Equilibrium* method is highly significantly better than the *Full* method and the *Average* method.

According to Table 2, each method has advantages and disadvantages. The gold-standard *Average* method does not need an estimation or expensive sensor hardware and has the lowest calibration time of all methods. Furthermore, the *Average* method still performs as the second best method in reducing muscle activity and as the method with the lowest intersubject variability. The *Full* method fails in the comparison due to higher estimation errors caused by a high number of inaccurate estimation parameters, including a freely estimated *CoM* in three dimensions. The *Full* method has the worst results and shows highest variability in the arm weight compensation efficacy comparison, although expensive sensors are needed. The novel *Equilibrium* method used the same costly sensor hardware to reach the significantly best result with the second lowest variability and a comparable low calibration time. All methods sufficiently fulfill the four ideal criteria set in this paper, as they provide freedom of movement, no additional large disturbances, scalability of upper and lower arm weight compensation, and a software solution that is applicable for all other actuated robots.

## Workspace analysis in stroke patients

### Study design: workspace analysis in stroke patients

**Fig. 6:**
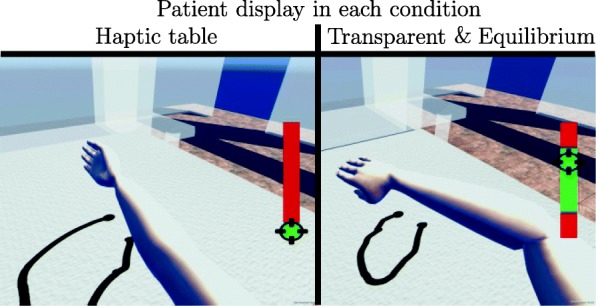
Patient display for the Haptic table, Transparent, and Equilibrium condition: The arm pose of the patient in ARMin was projected to the virtual avatar arm. The hand (end-effector) of the patient paints in black the boarders of the horizontal workspace on a virtual plane. The black target cursor in the red/green height panel shows the current height of the hand in the room. On the left side (haptic table), the patient can fully lean on the haptic table in the lower green panel part, which leads to passivity. Nevertheless, the haptic table condition provides a valuable measurement to show the physically maximal possible workspace for the patient. On the right side, the patient has to hold his/her arm actively in the middle green panel part, else the trial was ended and the patient was guided back to the starting pose

In the stroke patient study, the horizontal workspace of stroke patients was assessed at different height levels. In this way, the study protocol by Ellis et.al. [26] was extended to evaluate height-dependent arm weight compensation effects at the shoulder, chest, and abdomen height of each patient and to simultaneously test the developed arm weight compensation method in a feasibility study. The starting pose of the circular movement was chosen to be 40^∘^ shoulder flexion and 90^∘^ elbow flexion [26]. The patient was asked to perform the largest possible circular horizontal movement, which was repeated two times clockwise and counterclockwise. The evaluation was repeated under three loading conditions: With a haptic table [26], with the transparent robot without arm weight compensation (Transparent), and with the *Equilibrium* arm weight compensation method (Equilibrium). The three height levels (shoulder, chest, abdomen) were adapted to the individual anthropometry of the patient and the height levels were randomized block-wise for every patient. Concurrent visual feedback was provided for each test condition in order to obtain the correct height. For each condition, the movement direction of the two repetitions was block-wise randomized. After each movement, the patient could relax for approximately 30 s in the position-controlled start pose to minimize the influence of fatigue. To obtain a horizontal workspace measure, a vertical bar showed the allowed vertical range of the patient in all conditions (Fig. 6).

As shown in past studies [26], the horizontal workspace analysis is an appropriate method to assess the efficacy of arm weight compensation, since over- and undercompensation of arm weight always leads to further patient activity for doing corrections. If the patient’s arm was too high or too low, an additional arrow indicated to move the black height tracker from the red panel back into the green panel. If a maximum of 10 cm vertical deviation was exceeded, the trial was ended and the patient was moved to the starting pose by position control. Patient errors such as moving in the wrong direction would lead to a repetition of the trial. In the haptic table condition, the patient could fully rest on the virtual haptic object as indicated by the green area in the vertical height bar (Fig. 6).

### Primary outcomes: workspace analysis in stroke patients

The maximum horizontal workspace (in *m*^2^) for each condition (haptic table, Transparent, and *Equilibrium*) at each height level (shoulder, chest, and abdomen) reached with the summarized 2 circular clockwise and anti-clockwise measurements is the primary outcome of this study. The calculation of the visual maximum horizontal workspace was done with the “boundary” function in MATLAB.

### Subjects: workspace analysis in stroke patients

Three chronic stroke patients were recruited as subjects for the study. Their characteristics are presented in Table 3.

**Table 3:** Stroke patient characteristics for patients *P*_1_, *P*_2_, and *P*_3_ for the workspace analysis study and the estimated force parameters of method *Equilibrium*

Patients	*P*_1_	*P*_2_	*P*_3_
Age (years)	65	51	65
Gender (F/M)	F	F	M
Years after stroke	6	4	3
Dominant arm	Right	Right	Right
Affected arm	Right	Right	Right
Fugl-Meyer (max. 66)	26	-	39
Chedoke-McMaster	-	Arm: 3/7	-
		Hand 5/7	
Upper arm length (cm)	30.0	26.4	28.3
Lower arm length (cm)	29.7	29.9	29.6
Estimation force $\hat {f}_{ua}$ (N)	23.3	36.3	27.7
Estimation force $\hat {f}_{la}$ (N)	11.9	11.0	9.5

### Results: workspace analysis in stroke patients

**Fig. 7:**
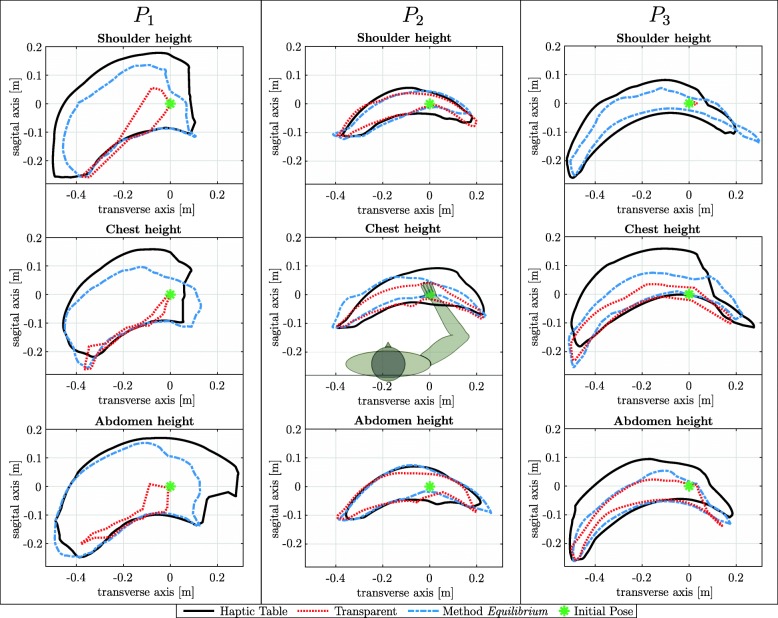
Maximum horizontal workspace of stroke patients on three different height levels: Maximum horizontal workspace of three stroke patients *P*_*i*_,*i*∈(1,2,3) on the three different horizontal height levels of abdomen height, chest height, and shoulder height. The haptic table condition (black line), the unsupported transparent condition (red dotted line), the novel arm weight compensation *Equilibrium* method condition (blue dashed line), and the initial hand pose (green star) are displayed

**Patient 1** The workspace in the transparent condition with maximum sagittal axis values of 0.04 *m* are closer to the body than for the *Equilibrium* method with a maximum value of 0.14 *m* (see Fig. 7). For the transparent condition, the initial position is the right-most point on the transverse axis. The area of the *Equilibrium* method condition is approximately 17% to 30% smaller than that of the haptic table condition (see Table 4).

**Table 4:** Maximum horizontal workspace relative to the Haptic Table condition in percent: The maximum horizontal workspace of the conditions Transparent and Equilibrium relative to condition Haptic Table (percentage %) are presented for each subject and height level

workspace normed to table condition in %		
	Shoulder height	
Patient	Transparent	*Equilibrium*
P1	13.9	69.8
P2	75.7	81.6
P3	0.2	56.5
	Chest height	
P1	7.4	82.7
P2	49.7	67.9
P3	32.4	54.7
	Abdomen height	
P1	8.2	78.4
P2	79.7	93.0
P3	34.8	53.8

Three stroke patients *P*_*i*_,*i*∈(1,2,3) on the three different horizontal levels (abdomen, chest, and shoulder height) are presented

**Patient 2** The total area covered for each condition is always approximately 0.04 *m*^2^ (see Fig. 7). In comparison with the other two patients, the area gains with arm weight compensation are lower (see Table 4).

**Patient 3** The area of the gravity condition decreases with table height (see Fig. 7). The weight compensation at least doubles the workspace of *P*_3_ for every table height (see Table 4). During arm weight compensation about 50% to 63% of the maximum reachable workspace of the haptic table scenario was reached. At the middle table height the most sagittal point 0.58*m* is reached in the haptic table scenario. The other two scenarios only reach a maximum sagittal axis value of approximately 0.51*m*.

### Discussion: workspace analysis in stroke patients

**General** The condition *Haptic Table* shows the maximum possible workspace for the patients at each height level. Patients can fully lean their arm on the haptic table. However, usage of the haptic table leads to passivity, reduced dimensionality, and no scalability [42]. Therefore, usage of a haptic table is not desired in rehabilitation training but can be used as patient-dependent maximum possible workspace reference for arm weight compensation methods.

**Patient 1** For arm weight compensation, patient *P*_1_ reached 0.1 *m* higher sagittal axis values and reached workspace gains of 500% to 1500% in comparison to the *Transparent* condition, which shows that the patient was able to extend the elbow more with arm weight compensation. The small differences from the maximum possible workspace in the haptic table condition in Table 4 underline that the patient could complete the given task with weight compensation to a similar quality as with a supportive haptic table.

**Patient 2** From the data and performance of patient *P*_2_ and visual observations during the experiment, it is likely that *P*_2_ was not able to cognitively understand the instructions or the task. The movements for each condition and scenario seemed to be similar by visual inspection of Fig. 7. In addition, the weight compensation and the virtual haptic table did not lead to important differences in the horizontal workspace. *P*_2_ likely moved on a preplanned movement trajectory independent of the transparent, *Equilibrium* method, or haptic table condition. At least the test with this patient showed the feasibility of all conditions and, in particular, the calibration of the arm weight compensation *Equilibrium* method.

**Patient 3** Patient *P*_3_’s workspace showed clearly height-dependent effects, as the patient did not improve with arm weight compensation at the abdomen level. In the shoulder height condition the patient was not able to hold the arm with own power against gravity. Nevertheless, with weight compensation or the haptic table, the patient was able to perform movements similar to those showed on the abdominal height. Similar to *P*_1_, *P*_3_ could extend the elbow in the weight compensation and haptic table scenario more in sagittal axis direction.

**Overview** We analyzed the height-dependent effects of workspace size related to weight compensation in patients. While previously reported effects of arm weight compensation (increased workspace) [26] could be confirmed, we also saw that patient-specific effects on the workspace size were present dependent on arm height level. Each patient showed different workspaces in the haptic table condition on different height levels. Higher height level implied for patient *P*_1_ loss of the workspace on the positive side of the transverse axis. For patient *P*_3_, both extreme height levels (shoulder, abdomen) caused a decrease of workspace on the sagittal axis. Individual height-dependent effects were also present in the transparent condition, which showed the patients’ capabilities without support. Patient *P*_3_ showed a clear loss of workspace for higher height levels and patient *P*_1_ maintained the workspace on both extreme height levels. Overall the *Equilibrium* arm weight compensation method always reached the maximum possible workspace (haptic table condition) to a high percentage, underlining its successful performance. While three subjects do not allow to draw general conclusions, this study showed the feasibility of our arm weight compensation method Equilibrium in a first place and further indicates that height dependence in weight compensation requires further investigation.

## General discussion

Quantitative evaluation and high efficacy of human arm weight compensation are crucial for the quality of assessment, individuality, and intensity of training that can be provided to patients. The evaluation presented in this paper contains a sensitivity analysis (for understanding the robustness of the estimation to estimation poses and time), efficacy comparison of different arm weight compensation methods (for understanding the performance implications of different methods), and a workspace analysis in stroke patients (to show the applicability of the best performing method in the target population).

Not all weight compensation methods available on the market can achieve a highly efficient arm weight compensation against gravity. For example, in passive mechanical devices that use spring-based elements to support the human arm against gravity, the support level is not the same in every arm pose [12]. Robotic devices with actuation can use human arm models that utilize sensor information (e.g. arm pose) to achieve a more consistent arm weight compensation efficacy independent of the arm pose. However, end-effector robots mostly can neither track nor actuate the upper and lower arm separately. Therefore, the exact pose-dependent torque to compensate arm weight is not applicable. On the other hand, for the exoskeleton robots, arm weight compensation is limited by the number of degrees of freedom with respect to the human arm.

With the ARMin rehabilitation exoskeleton, we provide arm weight compensation using five degrees of freedom (shoulder elevation, shoulder horizontal abduction, shoulder rotation, elbow flexion/extension, lower arm pronation/supination) that allows sufficient compensation against gravity in most arm poses. Three methods according to the four introduced criterions for ideal arm weight compensation have been introduced and evaluated: freedom of movement of human arm joints in the robot, no additional disturbances due to the method, independent scalability of upper and lower arm support, and applicability to other actuated systems. The methods can either approximate the arm weight using anthropometric tables (e.g.*Average* method) or estimate it for each subject (e.g. *Full* and *Equilibrium* methods).

Since the estimation accuracy affects the efficacy of the arm weight compensation, the sensitivity estimation study was conducted to analyze how the estimation pose and the time affect the model parameters, and to find the best pose for estimation. Temporal effects during estimation, therefore the estimation time, had no relevant influence on the estimation quality. On the other side, the estimation results are affected by the arm pose, probably due to the passive joint torques of the human arm and the misalignment effects between the arm and the robot. Therefore, care has to be taken to choose an estimation pose that will minimize these effects for the highest weight compensation efficacy.

Furthermore, the efficacy comparison of the arm weight compensation methods study highlighted a systematic way of evaluating the efficacy of any arm weight compensation method and showed that all three presented methods are successful in removing the arm weight of the subjects. It follows, that low-cost solutions that do not need force sensors like the current gold standard, the *Average* method, are a feasible solution for arm weight compensation. However, the *Equilibrium* method was significantly better at reducing the EMG activity of the subjects than the *Average* and *Full* methods. The performance of the *Equilibrium* method underlines that with estimated arm weight data acquired by force sensors, higher efficacy of arm weight compensation can be reached. The superiority of the *Equilibrium* to *Average* method is mainly due to having a parameter estimation phase. Nevertheless, parameter estimation with force sensors does not guarantee better performance than the gold standard *Average* method. The method *Full* had the highest remaining EMG activity because of having too many parameters to estimate and its estimation being very sensitive to the forces/torques caused by passive joint torques and misalignment between the human arm and robot axes. Having fewer parameters to estimate solves this problem as it can be seen with the *Equilibrium* method. Since the data used for parameter estimation is limited by the duration of the calibration (or estimation) phase, the simple model (*Equilibrium*) has less variance in estimation results and therefore is more robust to the estimation errors.

Finally, the *Equilibrium* method was tested in the workspace analysis study with stroke patients. The horizontal workspace evaluation study design of Ellis et.al. [26] was extended to evaluate height-dependent arm weight compensation effects at the shoulder, chest, and abdomen height of each patient and to simultaneously test the developed arm weight compensation method in a feasibility study. Our results confirm those from Ellis et.al. [26] : Horizontal workspace increases with arm weight compensation. Therefore, our results also highlight the importance of arm weight compensation for robot-assisted stroke rehabilitation. Different height-dependent effects in all three tested height conditions underline the need for individualized and scalable arm weight compensation in therapy.

## Conclusion

Three arm weight compensation methods were developed according to initially defined ideal arm weight compensation criteria, analyzed for temporal and spatial sensitivity of the resulting estimations, and compared using the ARMin rehabilitation robot towards their efficacy in reducing EMG activity in healthy subjects over the entire arm workspace. All three methods decreased the mean EMG activity to at least 49%. The force/torque sensor-based *Equilibrium* method performed significantly better than all other methods, including the *Average* method, which employs the current gold standard of using anthropometric table data for arm weight compensation, and the *Full* method, which is an arm model estimation method that employs force/torque sensors.

The horizontal workspace size evaluation with the best arm weight compensation method *Equilibrium* in stroke patients validates and expands upon previous studies by a qualitative evaluation at shoulder, chest, and abdomen height, suggesting individual height-dependent effects.

## Supplementary information

**Additional file 1** Arm Weight Compensation Methods.

**Additional file 2** Sensitivity Study.

## Data Availability

The dataset used and/or analysed during the current study are available from the corresponding author upon reasonable request.
