# Supplementary material for: Human arm weight compensation in rehabilitation robotics: efficacy of three distinct methods
Source: J Neuroeng Rehabil. 2020 Feb 5;17:13. doi: 10.1186/s12984-020-0644-3 (PMC7003349; doi:10.1186/s12984-020-0644-3)
Supplement: Supplementary file 1 — Additional file 1 Arm Weight Compensation Methods. [file 12984_2020_644_MOESM1_ESM.pdf]

RESEARCH

Additional File 1

Fabian Just[1*†], Özhan Özen[2†], Stefano Tortora[3], Verena Klamroth-Marganska[4], Robert Riener[1] and Georg Rauter[1,5]

Arm Weight Compensation Methods

Average Arm Method (Method *Average*)

Any arm attached to a robot can be accounted for in a model-based weight compensation. The required information to compensate weight in a quasi-static manner are the mass m of the arm and the location of its center of mass (CoM). This method is based on a model where the arm is assumed to consist of two segments, the upper arm and lower arm, which are rigidly connected to the robot (see Fig. 1).

The forces/torques created by the arm weight m and its according three-dimensional location vector $\boldsymbol{r}$ from CoM to the robot attachment cuffs is approximated by the 6 dimensional wrench vector

$$^{c}\tilde{\boldsymbol{\omega}}_{weight} = \begin{bmatrix} \boldsymbol{R}_0^c \; {}^0\tilde{\boldsymbol{f}} \\ {}^c\tilde{\boldsymbol{r}} \times \boldsymbol{R}_0^c \; {}^0\tilde{\boldsymbol{f}} \end{bmatrix}. \qquad (1)$$

The approximated weight vector ${}^0\tilde{\boldsymbol{f}} = \left[0, 0, \tilde{f} = -\tilde{m}g\right]^T$ is represented in the earth frame (x_0, y_0, z_0). Therefore, the approximated weight $\tilde{m}$ and the gravitational constant g are in z_0 direction. Vector ${}^c\tilde{\boldsymbol{r}}$ is the approximated $\boldsymbol{r}$ vector at each cuff frame c at the upper arm, $(c = c_{ua})$, and the lower arm, $(c = c_{la})$, respectively (see Fig. 1). Finally, $\boldsymbol{R}_0^c$ is the rotation matrix that rotates any vector in the earth frame to the cuff frame. This transformation is needed to represent the $\boldsymbol{r}$ vector with respect to the cuff frame. Please note that the resulting wrench vector ${}^c\tilde{\boldsymbol{\omega}}_{weight}$ is also represented in the cuff frame.

The parameters of the *Average* method for the upper and lower arm (masses $\tilde{m}_{ua}$, $\tilde{m}_{la}$ and center of mass ($\tilde{CoM}$) location vectors $\tilde{\boldsymbol{r}}_{ua}, \tilde{\boldsymbol{r}}_{la}$) are approximated using anthropometric tables of the average human [1]. These tables provide an approximation of lower and upper arm masses and the location of each CoM based on the subject's height and weight. Once the four parameters ($\tilde{m}$ and the three spatial $\tilde{\boldsymbol{r}}$ parameters:) are approximated, the ${}^c\tilde{\boldsymbol{\omega}}_{weight}$ is fully defined and the compensation torque for each respective arm segment, $\boldsymbol{\tau}_c$, is:

$$\boldsymbol{\tau}_c = -{}^c\boldsymbol{J}_c^T \; {}^c\tilde{\boldsymbol{\omega}}_{weight} = -{}^0\boldsymbol{J}_c^T \; \boldsymbol{R}_c^0 \; {}^0\tilde{\boldsymbol{\omega}}_{weight}, \qquad (2)$$

where ${}^c\boldsymbol{J}_c^T$ and ${}^0\boldsymbol{J}_c^T$ are the Jacobians of the origin of the cuff represented in the cuff and earth frame, respectively, and $\boldsymbol{R}_c^0$ is the rotation matrix, which rotates vectors in cuff frame to the earth frame. Finally, the provided arm weight compensation, $\boldsymbol{\tau}_{comp}$, is the sum of upper and lower arm weight compensation torques:

$$\begin{aligned} \boldsymbol{\tau}_{comp} = &- {}^{c_{ua}}\boldsymbol{J}_{c_{ua}}^T \; {}^{c_{ua}}\tilde{\boldsymbol{\omega}}_{weight,ua} \\ &- {}^{c_{la}}\boldsymbol{J}_{c_{la}}^T \; {}^{c_{la}}\tilde{\boldsymbol{\omega}}_{weight,la}. \end{aligned} \qquad (3)$$

Note that this procedure obtains the same result as other methods, similar to creating a Jacobian for the position of the CoM of the object and using direct multiplication with the augmented 6 dimensional $\tilde{\boldsymbol{f}}$ vector. However, this is not mathematically straightforward because the arm segments are connected to the robot from the cuffs, not from the $CoMs$. To adapt the parameters to each subject, the therapists must manually enter height and weight information for every new subject, which requires approximately half a minute.

The herein proposed model-based arm weight compensation uses the same principle as weight compensation of rigid structure segments within the robot. The upper arm and lower arm are assumed to be two independent rigid bodies attached to the exoskeleton structure of the robot. That means this approach relies on the assumption that the upper and lower arms are not interacting with each other [2]. The effect of this assumption on the performance of the weight compensation is analysed in the main text.

Full Estimation Method (Method *Full*)

The *Full* method uses a model assuming that the arm consists of the two separated segments, the upper arm and lower arm, which are rigidly connected to the robot. All the parameters of the method ($\hat{m}$, $\hat{r}_x$, $\hat{r}_y$, $\hat{r}_z$) are assumed to be nonzero (see Fig. 1). Compared

*Correspondence: fabian.just@hest.ethz.ch
[1]Sensory-Motor Systems (SMS) Lab, Institute of Robotics and Intelligent Systems (IRIS), Department of Health Sciences and Technology (D-HEST), ETH Zurich, Switzerland and Reharobotics Group, Spinal Cord Injury Center, Balgrist University Hospital, Medical Faculty, University of Zurich, Switzerland, Lengghalde 5, 8092 Zurich, Switzerland
Full list of author information is available at the end of the article
†Fabian Just and Özhan Özen contributed equally to this work.

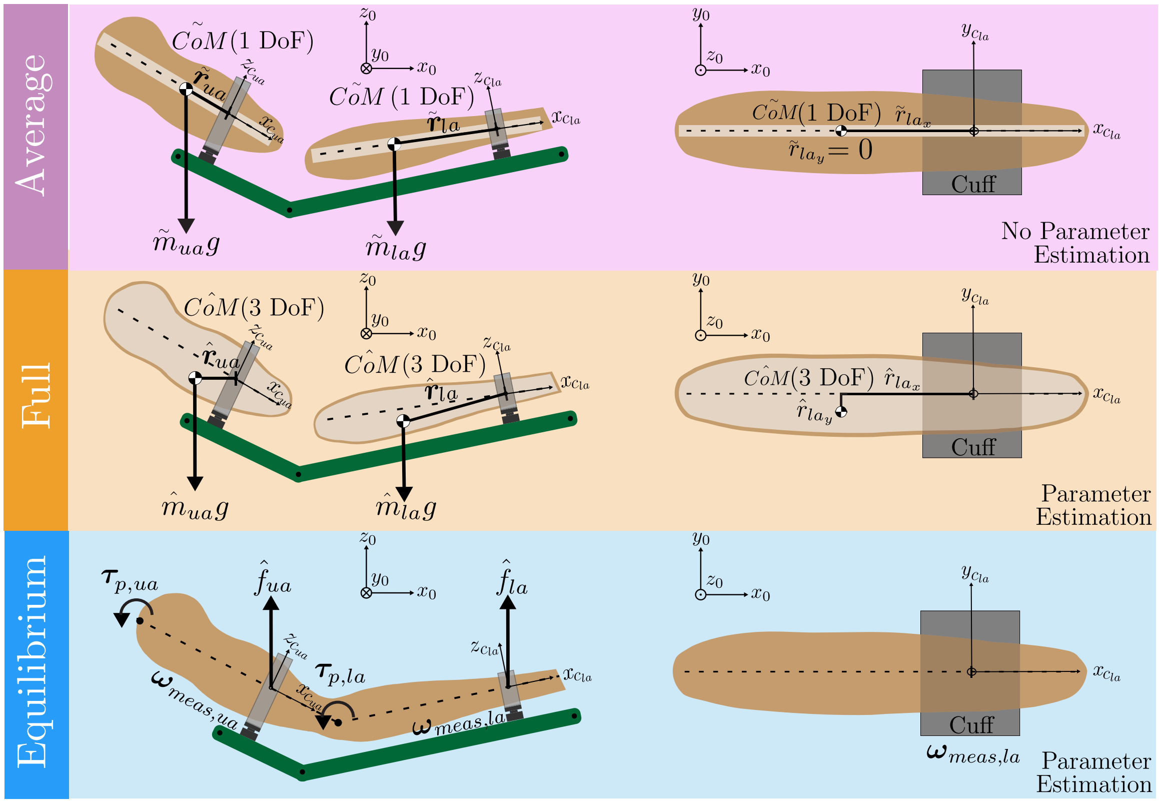

Figure 1 The three arm weight compensation methods are illustrated in side view and top view.
Method *Average*: Masses $\tilde{m}$ and the center of masses ($\tilde{CoM}$) are calculated through anthropometric tables with subject data. The arm is modeled as two independent rigid bodies, upper arm (ua) and lower arm (la). The $CoMs$ of the two arm segments are assumed to lay on the dashed arm segment rotation axis. Therefore, only 1DoF of the CoM is adapted with the antropometric table information.
Method *Full*: Similar to Method *Average*, the arm is modeled as two independent rigid bodies. However, $CoMs$ of the objects are not assumed to lay on the rotation axis. The arm parameters $\hat{m}$ and the 3 DoF $\hat{CoM}$ are estimated using force/torque sensors.
Method *Equilibrium*: An equilibrium against gravity is created through force/torque sensor data at each cuff. Upper and lower arm are assumed to be connected and the passive joint torques $\boldsymbol{\tau}_{p,ua}$ and $\boldsymbol{\tau}_{p,la}$ are taken into account. Finally, the support forces $\hat{f}_{ua}$ and $\hat{f}_{la}$ are calculated and achieve an equilibrium against the measured wrenches created by gravity at the cuffs, $\omega_{meas,ua}$,$\omega_{meas,la}$. The $CoMs$ of the arm are assumed to lie on each arm segment rotation axis and are not calculated compared to the two other methods.

with the *Average* method in (1) all parameters of the *Full* method are estimated with force/torque sensor data and not approximated,

$$^{c}\hat{\boldsymbol{\omega}}_{weight} = \begin{bmatrix} \boldsymbol{R}_0^c \; {}^0\hat{\boldsymbol{f}} \\ {}^c\hat{\boldsymbol{r}} \times \boldsymbol{R}_0^c \; {}^0\hat{\boldsymbol{f}} \end{bmatrix}. \tag{4}$$

The estimated weight vector ${}^0\hat{\boldsymbol{f}} = \left[0, 0, \hat{f} = -\hat{m}g\right]^T$ is represented in the earth frame (x_0, y_0, z_0). Therefore, the estimated mass $\hat{m}$ and the gravitational constant g are in the z_0 direction. The vector ${}^c\tilde{\boldsymbol{r}}$ is the estimated $\boldsymbol{r}$ vector at each cuff frame c of the upper arm, $(c = c_{ua})$, and the lower arm, $(c = c_{la})$, respectively. Finally, the arm weight compensation torque $\boldsymbol{\tau}_{comp}$ is the sum of the upper and lower arm compensation torques,

$$\begin{aligned} \boldsymbol{\tau}_{comp} = & - {}^{c_{ua}}\boldsymbol{J}_{c_{ua}}^T \; {}^{c_{ua}}\hat{\boldsymbol{\omega}}_{weight,ua} \\ & - {}^{c_{la}}\boldsymbol{J}_{c_{la}}^T \; {}^{c_{la}}\hat{\boldsymbol{\omega}}_{weight,la}, \end{aligned} \tag{5}$$

where ${}^{c_{ua}}\boldsymbol{J}_{c_{ua}}^T$ and ${}^{c_{la}}\boldsymbol{J}_{c_{la}}^T$ are the Jacobians that project the respective wrench vector on the joint axes. During the initial estimation of all parameters, which occurs once during therapy, it is necessary that the subject does not apply any force and completely leaves his/her arm on the robot in a fixed pose. The procedure is as follows: The rotation matrix for any cuff frame c at any arbitrary pose (θ_k) can be represented

as:

$$\boldsymbol{R}_0^c(\theta_k) = \begin{bmatrix} r_{11}(\theta_k) & r_{12}(\theta_k) & r_{13}(\theta_k) \\ r_{21}(\theta_k) & r_{22}(\theta_k) & r_{23}(\theta_k) \\ r_{31}(\theta_k) & r_{32}(\theta_k) & r_{33}(\theta_k) \end{bmatrix} \quad (6)$$

Substituting (6) and (4) and, replacing ${}^c\hat{\boldsymbol{\omega}}_{weight}$ with the measured wrench at the cuff ${}^c\boldsymbol{\omega}_{meas}(\theta_k)$ during measurement at the arbitrary pose (θ_k) yields the following equality:

$$\begin{bmatrix} -\hat{m}g\ r_{13}(\theta_k) \\ -\hat{m}g\ r_{23}(\theta_k) \\ -\hat{m}g\ r_{33}(\theta_k) \\ \hat{m}g\ r_{23}(\theta_k)\ \hat{r}_z\ -\ \hat{m}g\ r_{33}(\theta_k)\ \hat{r}_y \\ \hat{m}g\ r_{33}(\theta_k)\ \hat{r}_x\ -\ \hat{m}g\ r_{13}(\theta_k)\ \hat{r}_z \\ \hat{m}g\ r_{13}(\theta_k)\ \hat{r}_y\ -\ \hat{m}g\ r_{23}(\theta_k)\ \hat{r}_x \end{bmatrix} = \begin{bmatrix} f_x(\theta_k) \\ f_y(\theta_k) \\ f_z(\theta_k) \\ \tau_x(\theta_k) \\ \tau_y(\theta_k) \\ \tau_z(\theta_k) \end{bmatrix}_{meas} \quad (7)$$

which results in a system of six scalar equations. In theory, the first three equations, which are linear, are not independent, and thus, one of them is sufficient to estimate the arm weight $\hat{m}$ of the arm. Once the estimated arm weight $\hat{m}$ is know, the remaining three equations become linear equations too. However, these equations are not enough to solve for $\hat{r}_x$, $\hat{r}_y$ and $\hat{r}_z$ due to the fact that the columns of the rotation matrices are constrained to be an unit vector:

$$r_{13}(\theta_k)^2 + r_{23}(\theta_k)^2 + r_{33}(\theta_k)^2 = 1. \quad (8)$$

Due to this constraint, two measurements at two different estimation poses (θ_1 and θ_2) must be made. The resulting equation system with 12 equations is sufficient to solve for all variables.

Previously, we have assumed that the first three equations should theoretically provide the same value for the estimated arm weight $\hat{m}$, independently from the estimation pose. However, due to practical inaccuracies of the force/torque sensors and noise, they provide different results for the mass estimation, $\hat{m}$. To minimize this stochastic error, the least squares method was used. The over determined system summarized from the two measurement poses (θ_1 and θ_2) to apply the least squares method is as follows:

$$\begin{bmatrix} -g\ r_{13}(\theta_1) \\ -g\ r_{23}(\theta_1) \\ -g\ r_{33}(\theta_1) \\ -g\ r_{13}(\theta_2) \\ -g\ r_{23}(\theta_2) \\ -g\ r_{33}(\theta_2) \end{bmatrix} [\hat{m}] = \begin{bmatrix} f_x(\theta_1) \\ f_y(\theta_1) \\ f_z(\theta_1) \\ f_x(\theta_2) \\ f_y(\theta_2) \\ f_z(\theta_2) \end{bmatrix}_{meas} . \quad (9)$$

After the arm weight $\hat{m}$ has been found, the CoM location vector $\hat{\boldsymbol{r}}$ values in each direction need to be computed by applying least squares to the following equations composed of the two measurement poses (θ_1 and θ_2):

$$\begin{bmatrix} 0 & -\hat{m}g\ r_{33}(\theta_1) & \hat{m}g\ r_{23}(\theta_1) \\ \hat{m}g\ r_{33}(\theta_1) & 0 & -\hat{m}g\ r_{13}(\theta_1) \\ -\hat{m}g\ r_{23}(\theta_1) & \hat{m}g\ r_{13}(\theta_1) & 0 \\ 0 & -\hat{m}g\ r_{33}(\theta_2) & \hat{m}g\ r_{23}(\theta_2) \\ \hat{m}g\ r_{33}(\theta_2) & 0 & -\hat{m}g\ r_{13}(\theta_2) \\ -\hat{m}g\ r_{23}(\theta_2) & \hat{m}g\ r_{13}(\theta_2) & 0 \end{bmatrix} \begin{bmatrix} \hat{r}_x \\ \hat{r}_y \\ \hat{r}_z \end{bmatrix} = \begin{bmatrix} \tau_x(\theta_1) \\ \tau_y(\theta_1) \\ \tau_z(\theta_1) \\ \tau_x(\theta_2) \\ \tau_y(\theta_2) \\ \tau_z(\theta_2) \end{bmatrix}_{meas} \quad (10)$$

Once the four parameters $\hat{m}$, $\hat{r}_x$, $\hat{r}_y$ and $\hat{r}_z$ are identified, they can be used in (5) for the calculation of the arm weight compensation torque, $\boldsymbol{\tau}_{comp}$.

Equilibrium Estimation Method (Method *Equilibrium*)

In reality, the anatomical joints connecting human limbs cannot be considered ideal joints, and passive joint torques caused between body segments or through the twisting of body segments in the cuffs cannot be considered negligible [3]. The unintentional passive joint torques created by the shoulder on the upper arm, $\boldsymbol{\tau}_{p,ua}$, and the passive torques created by the upper arm on the lower arm, $\boldsymbol{\tau}_{p,la}$, may not be small enough to be neglected. The *Equilibrium* method partially considers passive joint torques as part of the measured interaction wrench at the upper ($\boldsymbol{\omega}_{meas,ua}$) and lower arm ($\boldsymbol{\omega}_{meas,ua}$) during calibration.

We first consider the case with ARMin (or any similar arm exoskeleton robot) for which the pose of the robot is fixed, and the arm inside is in static equilibrium (see Fig. 1). The cuffs where the arm is attached apply support forces at the contact points in the negative gravity direction. Indeed, for the upper and lower arm to be in static equilibrium, these estimated support forces, $\hat{f}_{ua}$ and $\hat{f}_{la}$, should have a projection in the negative gravity direction. Please note that the cuffs are assumed not to apply torques in the y_0 direction by design, and the friction is neglected.

If the summarized passive joint torques are assumed to point in y_0 direction and are similar for every possible arm position, then the estimated support forces, $\hat{f}_{ua}$ and $\hat{f}_{la}$, which are required to hold the arm in

static equilibrium would be constant, and independent of the pose. The method *Equilibrium* constructs two estimated support force parameters at the arm cuffs, which should realize a static equilibrium for the arm independent of the pose, if no other intentional force is applied. Applied on the joint torques the arm weight compensation torque $\boldsymbol{\tau}_{comp}$ is a product of the wrench vector including the force parameter and of the respective Jacobian projecting from the global coordinate system at the joint level:

$$\boldsymbol{\tau}_{comp} = {}^0\boldsymbol{J}_{c_{ua}}^T \begin{bmatrix} 0 \\ 0 \\ \hat{f}_{ua} \\ 0 \\ 0 \\ 0 \end{bmatrix} + {}^0\boldsymbol{J}_{c_{la}}^T \begin{bmatrix} 0 \\ 0 \\ \hat{f}_{la} \\ 0 \\ 0 \\ 0 \end{bmatrix}. \tag{11}$$

The method utilizes an torque equilibrium with the torques induced by the weight of the passive arm measured through the force/torque sensors during calibration, $\boldsymbol{\tau}_{meas}$, in a position controlled robot pose, for the estimation of the support forces, $\hat{f}_{ua}$ and $\hat{f}_{la}$,

$$\boldsymbol{\tau}_{meas} = {}^0\boldsymbol{J}_{c_{ua}}^T\, {}^0\boldsymbol{\omega}_{meas,ua} + {}^0\boldsymbol{J}_{c_{la}}^T\, {}^0\boldsymbol{\omega}_{meas,la}. \tag{12}$$

The torque vector $\boldsymbol{\tau}_{meas}$ is the product of the measured interaction wrenches at the physical human-robot interaction points at the upper (${}^0\boldsymbol{\omega}_{meas,ua}$) and the lower arm (${}^0\boldsymbol{\omega}_{meas,la}$) and of their respective Jacobian in the earth coordinate frame projecting on the robot joints. The position of the $CoMs$ or the masses of the arms are not considered to be independent. In the end, the torques created by the weight of the arm, $\boldsymbol{\tau}_{meas}$, should be equal (but with opposite signs) to the torques created by these virtual support forces, such that the weight of the arm is compensated. The estimation scheme is based on this idea, reducing the number of parameters to estimate from eight to two compared to method *Full* (from two mass and six CoM location vector parameters to two support force parameters).

If the assumptions hold, the following relation is valid for a passive subject and used to estimate the force parameters $\hat{f}_{ua}$ and $\hat{f}_{la}$:

$$\boldsymbol{\tau}_{meas} + {}^0\boldsymbol{J}_{c_{ua}}^T \begin{bmatrix} 0 \\ 0 \\ \hat{f}_{ua} \\ 0 \\ 0 \\ 0 \end{bmatrix} + {}^0\boldsymbol{J}_{c_{la}}^T \begin{bmatrix} 0 \\ 0 \\ \hat{f}_{la} \\ 0 \\ 0 \\ 0 \end{bmatrix} = 0. \tag{13}$$

Where ${}^0\boldsymbol{J}_{c_{ua}}^T$ and ${}^0\boldsymbol{J}_{c_{la}}^T$ are the Jacobians of the origin of the sensors represented in earth frame. Mathematical manipulations of equation (13) lead to the following equation:

$$\begin{bmatrix} j_{c_{ua}}(1,3) & j_{c_{la}}(1,3) \\ j_{c_{ua}}(2,3) & j_{c_{la}}(2,3) \\ \vdots & \vdots \\ j_{c_{ua}}(n,3) & j_{c_{la}}(n,3) \end{bmatrix} \begin{bmatrix} \hat{f}_{ua} \\ \hat{f}_{la} \end{bmatrix} = \begin{bmatrix} \tau_{meas}(1) \\ \tau_{meas}(2) \\ \vdots \\ \tau_{meas}(n) \end{bmatrix} \tag{14}$$

where $j_{c_{ua}}$ and $j_{c_{la}}$ are the elements of the Jacobians of the upper and lower arm cuff as seen in equation (13), and n is the number of axes of rotations of the robot up to the lower arm. For the ARMin exoskeleton five axes of rotation, $n = 5$, are included, and since this value is larger than two ($n > 2$) even for a single pose, the linear equation system is overdetermined and can be solved by a least squares method. Note that, since the support forces are in the z_0 direction of the earth frame, only the multiplication with the third column variables of the Jacobian will result in nonzero values.

Here, the ordinary least squares method (using the same weight for each equation) may be prone to sensor noise/errors in practise. Thus, we consider the force sensor error projected to the axis resulting from (14):

$$\begin{gathered} j_{c_{ua}}(x,3)\ \hat{f}_{ua} + j_{c_{la}}(x,3)\ \hat{f}_{la} = \\ \tau_{meas}(x) + \tau_{meas_{error}}(x), \\ x = 1, ..., 5 \end{gathered} \tag{15}$$

Due to the pose where the estimation is made, $j_{c_{ua}}(x,3)$ and $j_{c_{la}}(x,3)$ can approach zero along with the $\tau_{meas}(x)$. In such scenarios, $\tau_{meas}(x)$ dominates the right-hand-side and the estimation contribution from this equation may cause a huge error.

To prevent this, a weighted least squares method is used, where the weights are selected according to the Jacobian values, such that:

$$w(x) = \frac{|j_{c_{ua}}(x,3)| + |j_{c_{la}}(x,3)|}{w(1) + w(2) + ... + w(5)},\ where\ x = 1, 2, ...5 \tag{16}$$

This way, the equations that are most likely to lead to huge errors are considered less in the estimation. The weighted least squares solution is then calculated by:

$$\begin{bmatrix} \hat{f}_{ua} \\ \hat{f}_{la} \end{bmatrix} = (\boldsymbol{A}^T\boldsymbol{W}\boldsymbol{A})^{-1}\boldsymbol{A}^T\boldsymbol{W}\boldsymbol{b} \tag{17}$$

With the elements:

$$\boldsymbol{A} = \begin{bmatrix} j_{c_{ua}}(1,3) & j_{c_{la}}(1,3) \\ j_{c_{ua}}(2,3) & j_{c_{la}}(2,3) \\ \vdots & \vdots \\ j_{c_{ua}}(n,3) & j_{c_{la}}(n,3) \end{bmatrix}, \ \boldsymbol{b} = \begin{bmatrix} \tau_{meas}(1) \\ \tau_{meas}(2) \\ \vdots \\ \tau_{meas}(n) \end{bmatrix},$$

$$\boldsymbol{W} = diag(w).$$

Please note that the estimation above requires only knowledge of the projection of the weight of the arm to the robot joints rather than wrenches at the cuff sensor points. This makes it possible to use Reaction Torque Observers (RTOB) for estimation and removes the need for the use of force sensors, which can be useful for robots that do not have force or force/torque sensors [4]. Note that, although this is the case, force/torque sensors are still used for the experiments of this paper. The used exoskeleton ARMin, has considerable static (break-away) friction, which is difficult to model. Complex robot models are prone to errors, which would reduce the RTOB performance [5].

Author details
[1]Sensory-Motor Systems (SMS) Lab, Institute of Robotics and Intelligent Systems (IRIS), Department of Health Sciences and Technology (D-HEST), ETH Zurich, Switzerland and Reharobotics Group, Spinal Cord Injury Center, Balgrist University Hospital, Medical Faculty, University of Zurich, Switzerland, Lengghalde 5, 8092 Zurich, Switzerland. [2]Motor Learning and Neurorehabilitation Laboratory, ARTORG Center for Biomedical Engineering Research, University of Bern, Freiburgstrasse 3, 3010 Bern, Switzerland. [3]IAS-Lab, Department of Information Engineering, University of Padova, via Giovanni Gradenigo 6a, 35131 Padova, Italy. [4]Institute of Occupational Therapy, School of Health Professions, Zurich University of Applied Sciences, Technikumstrasse 81, 8401 Winterthur, Switzerland. [5]BIROMED-Lab, Department of Biomedical Engineering, University of Basel, Düsternbrooker Weg 20, 4123 Allschwil, Switzerland.

References

1. Winter, D.A.: Biomechanics and Motor Control of Human Movement, (2009). doi:10.1002/9780470549148. arXiv:0712.2824v3. http://doi.wiley.com/10.1002/9780470549148
2. Just, F., Özen, Ö., Tortora, S., Riener, R., Rauter, G.: Feedforward model based arm weight compensation with the rehabilitation robot ARMin. IEEE International Conference on Rehabilitation Robotics (July), 72–77 (2017). doi:10.1109/ICORR.2017.8009224
3. Marieb, E.N., Hoehn, K.: Human Anatomy & Physiology, pp. 1095–1099. Pearson Education, ??? (2007). doi:10.1007/BF00845519. 1110.4742
4. Šabanović, A., Ohnishi, K.: Motion Control Systems vol. 9781447166740, pp. 793–846. John Wiley & Sons (Asia) Pte Ltd, ??? (2011). doi:10.1002/9780470825754. http://link.springer.com/10.1007/978-1-4615-4431-9_2 http://doi.wiley.com/10.1002/9780470825754
5. Just, F., Özen, Ö., Bösch, P., Bobrovsky, H., Klamroth-Marganska, V., Riener, R., Rauter, G.: Exoskeleton transparency: feed-forward compensation vs. disturbance observer. at - Automatisierungstechnik **66**(12), 1014–1026 (2018). doi:10.1515/auto-2018-0069
