# Supplementary material for: Human arm weight compensation in rehabilitation robotics: efficacy of three distinct methods
Source: J Neuroeng Rehabil. 2020 Feb 5;17:13. doi: 10.1186/s12984-020-0644-3 (PMC7003349; doi:10.1186/s12984-020-0644-3)
Supplement: Supplementary file 2 — Additional file 2 Sensitivity Study. [file 12984_2020_644_MOESM2_ESM.pdf]

RESEARCH

Additional File 2

Fabian Just[1*†], Özhan Özen[2†], Stefano Tortora[3], Verena Klamroth-Marganska[4], Robert Riener[1] and Georg Rauter[1,5]

Sensitivity Study

Study design

The estimation results of the *Full* and *Equilibrium* methods are both based on measured force/torque sensor data and therefore the arm weight force $(\hat{f}_{ua}, \hat{f}_{la})$ and for the *Full* method additionally the center of mass location vector $(\hat{\boldsymbol{r}}_{ua}, \hat{\boldsymbol{r}}_{la})$ need to be estimated with the subject inside the ARMin. Since the estimation quality differs for different subject anthropometries and different estimation poses, an analysis of the estimation behavior to finally achieve higher and more robust estimation quality is needed. To analyze estimation behavior, two main characteristics were investigated: First, the temporal changes of the estimation results, called temporal sensitivity, was evaluated with the estimation results' standard deviation over time. Second, the influence of the estimation pose on the estimation result, called spatial sensitivity, was tested. The standard deviation of the estimation results over all spatial estimation poses for each subject is the outcome measure for the spatial sensitivity analysis. For clinical evaluation of the temporal and spatial sensitivities of the *Full* and *Equilibrium* methods, the subject's arm was driven by the position-controlled ARMin in $p = 27$ different poses. In each of these poses the weight of the arm was estimated over six seconds during each iteration with the $1800 Hz$ real time system. The $p = 27$ poses were defined to cover the functional arm ROM [1] adapted to the passive ROM of stroke patients (see Tab. 1).

Table 1 Pose coordinates of the sensitivity analysis and the method comparison study according to anatomical axes definitions [2]. Axes number correspondence: axis 1 (horizontal shoulder abduction/adduction), axis 2 (shoulder elevation), axis 3 (internal /external shoulder rotation), axis 4 (elbow flexion/extension), and axis 5 (forearm pronation/supination).

pose	axis (°)				
	1	2	3	4	5
1	-25	55	30	0	60
2	0	70	30	0	60
3	-25	70	30	0	60
4	-25	90	30	0	60
5	0	90	30	0	60
6	25	90	30	0	60
7	25	70	30	0	60
8	25	80	30	45	60
9	0	55	30	90	60
10	-45	55	90	100	0
11	-45	70	30	100	60
12	0	70	30	90	60
13	0	90	90	90	0
14 & P_1	-45	90	90	100	0
15	0	90	30	45	60
16	0	90	5	45	0
17	0	70	5	60	0
18	0	55	5	90	0
19	-45	55	5	90	0
20 & P_2	-45	70	5	60	0
21	-45	70	45	45	0
22	-45	55	45	45	0
23	0	60	45	90	0
24	0	70	45	60	0
25	0	70	5	45	0
26	25	70	5	45	0
27	-60	70	45	45	0
P_3	10	70	30	40	60
P_4	-15	65	15	50	0
P_5	-50	55	30	40	0

Primary Outcome

For the temporal sensitivity analysis of the methods *Full* and *Equilibrium* the standard deviations of the estimated arm weight compensation force $(\hat{f}_{ua}, \hat{f}_{la})$ were evaluated over all measurement points $(m = 10800)$ for all subject arms $(s = 10)$ and estimation poses $(p = 27)$ combinations. The following derivation based on the upper arm force parameter $\hat{f}_{ua}$ is exemplary for both arm segments. First the standard deviation over the six second measurement samples m is calculated for each subject s and pose p, $\sigma_{\hat{f}_{ua,s,p}}$. It shows the temporal influence during the specific measurement at pose p with subject s. As the most general primary outcome, the mean and standard deviation over all subjects and poses of $\sigma_{\hat{f}_{ua,s,p}}$ were used: $\mu_{\sigma_{\hat{f}_{ua}}}$ and $\sigma_{\sigma_{\hat{f}_{ua}}}$. For a more specific temporal analysis of inter-pose variability, $\mu_{\sigma_{\hat{f}_{ua,p}}}$ and $\sigma_{\sigma_{\hat{f}_{ua,p}}}$ as the mean and standard deviation of $\sigma_{\hat{f}_{ua,s,p}}$ over all subjects were used to identify unstable estimation poses over time. Through the need of a pose-pair for method *Full*, $p = \binom{27}{2} = \frac{27!}{2!(27-2)!} = 351$ possible pose combinations were tested and compared with the *Equilibrium*

*Correspondence: fabian.just@hest.ethz.ch
[1]Sensory-Motor Systems (SMS) Lab, Institute of Robotics and Intelligent Systems (IRIS), Department of Health Sciences and Technology (D-HEST), ETH Zurich, Switzerland and Reharobotics Group, Spinal Cord Injury Center, Balgrist University Hospital, Medical Faculty, University of Zurich, Switzerland, Lengghalde 5, 8092 Zurich, Switzerland
Full list of author information is available at the end of the article
†Fabian Just and Özhan Özen contributed equally to this work.

method with the single $p = 27$ possible estimation poses. In addition to the *Equilibrium* method, the *Full* method estimates not only the arm weight compensation forces $\hat{\boldsymbol{f}}$ but also the center of masses ($CoMs$) location vectors of the upper $\hat{r}_{ua}$ and lower arm $\hat{r}_{la}$, which are also temporally analysed in the presented way.

After finalizing the temporal sensitivity analysis a mean value for all m measurement points at each pose and for each subject is taken for further pose-depending analysis. The primary outcome of the spatial sensitivity analysis is the average and standard deviation of the estimated parameter standard deviation ($\sigma_{\hat{f}_{ua,p}}$, $\sigma_{\hat{f}_{ua,p}}$) for all subjects evaluated over the $p = 27$ arm poses. This way, the effect of the selected arm pose on the estimation parameters can be shown.

Since subject-dependent singular estimation poses can lead to unfeasible data and can induce a significant shift on mean estimation results, a developed algorithm identified estimation poses with minimum misalignment torques and more reliable performance over all subjects. As a first step all estimations that were within a threshold δ_F relative to the measured gravitational forces at the respective $(f_{ua,k}, f_{la,k})$ upper and lower arm force/torque sensor were identified. The *Equilibrium* method with one necessary estimation pose k has the following criteria:

$$\begin{aligned} \hat{f}_{ua,k} - f_{ua,k} &< \delta_f, \qquad \forall k \in [1, ..., 27] \\ \hat{f}_{la,k} - f_{la,k} &< \delta_f, \end{aligned} \tag{1}$$

For the *Full* method the equivalent criteria include the estimation pose-pair (k,l). Additionally, a threshold criterion δ_r for the estimated CoM location vector of the upper and lower arm ($\hat{\boldsymbol{r}}_{ua}$, $\hat{\boldsymbol{r}}_{la}$) was introduced:

$$\begin{gathered} \hat{f}_{ua,k,l} - f_{ua,k} < \delta_f \quad \& \quad \hat{f}_{ua,k,l} - f_{ua,l} < \delta_f \\ \hat{f}_{la,k,l} - f_{la,k} < \delta_f \quad \& \quad \hat{f}_{la,k,l} - f_{la,l} < \delta_f \\ \hat{r}_{ua,x} < \delta_r \quad \& \quad \hat{r}_{ua,y} < \delta_r \quad \& \quad \hat{r}_{ua,z} < \delta_r \\ \hat{r}_{la,x} < \delta_r \quad \& \quad \hat{r}_{la,y} < \delta_r \quad \& \quad \hat{r}_{la,z} < \delta_r \\ \forall k, l \in [1, ..., 27], k \neq l \end{gathered} \tag{2}$$

The following algorithm steps are similar for both methods. For subject i, only the estimation pose k or the estimation pose-pair (k, l) that fulfills all of the respective criteria are parts of a reference arithmetic mean for the upper and lower arm

$$\overline{\hat{\boldsymbol{f}}}_{ref,k,i} = \left[\overline{\hat{f}}_{ref,ua,k,i}, \overline{\hat{f}}_{ref,la,k,i}\right]. \tag{3}$$

The difference from the reference $\overline{\hat{\boldsymbol{f}}}_{ref,k,i}$ for all estimations in a single subject is taken as an error measure

$$\hat{f}_{err,k,i} = \left|\overline{\hat{f}}_{ref,ua,k,i} - \hat{f}_{ua,k,i}\right| + \left|\overline{\hat{f}}_{ref,la,k,i} - \hat{f}_{la,k,i}\right| \tag{4}$$

The error measure is summed up over all N subjects to find the pose with minimum error:

$$\hat{f}_{err,k} = \sum_{i=1}^{N} \hat{f}_{err,k,i}. \tag{5}$$

The pose k with minimum error $\hat{f}_{err,k}$ is chosen as the best estimation pose. The thresholds δ_f and δ_r are chosen with known measurements results to be as small as possible and large enough to generate an individual reference arithmetic mean for every subject i.

Subjects

The sensitivity analysis was performed with 5 healthy subjects (3 females; 5 right-handed; age: 24.50 ± 2.17 years; height: 177.50 ± 10.21m; weight: 69.67 ± 10.71 kg). Their left and right arm was taken for evaluation resulting in a total of 10 arm measurement sets. Inclusion criteria were older than 18 years of age, no serious medical or psychiatric disorders, no cybersickness, no pacemaker or other implanted electric devices and body weight less than 120 kg.

Results

The temporal sensitivity analysis focuses on the temporal changes over all m measurement points showed the following results for the *Full* method:

$$\begin{aligned} \mu_{\sigma_{\hat{f}_{ua}}} &= 0.34N, \quad \sigma_{\sigma_{\hat{f}_{ua}}} &&= 0.16N \\ \mu_{\sigma_{\hat{r}_{ua,x}}} &= 0.33m, \quad \sigma_{\sigma_{\hat{r}_{ua,x}}} &&= 7.24m \\ \mu_{\sigma_{\hat{r}_{ua,y}}} &= 0.14m, \quad \sigma_{\sigma_{\hat{r}_{ua,y}}} &&= 2.9m \\ \mu_{\sigma_{\hat{r}_{ua,z}}} &= 0.39m, \quad \sigma_{\sigma_{\hat{r}_{ua,z}}} &&= 12.7m \\ \mu_{\sigma_{\hat{f}_{la}}} &= 0.13N, \quad \sigma_{\sigma_{\hat{f}_{la}}} &&= 0.08N \\ \mu_{\sigma_{\hat{r}_{la,x}}} &= 5 \cdot 10^{-13}m, \quad \sigma_{\sigma_{\hat{r}_{la,x}}} &&= 1 \cdot 10^{-11}m \\ \mu_{\sigma_{\hat{r}_{la,y}}} &= 3 \cdot 10^{-13}m, \quad \sigma_{\sigma_{\hat{r}_{la,y}}} &&= 8 \cdot 10^{-12}m \\ \mu_{\sigma_{\hat{r}_{la,z}}} &= 4 \cdot 10^{-13}m, \quad \sigma_{\sigma_{\hat{r}_{la,z}}} &&= 9 \cdot 10^{-12}m \end{aligned} \tag{6}$$

Further evaluation of the pose-pair depending standard deviation of the CoM location vector characteristics, $\sigma_{\sigma_{\hat{r}_{ua,x,p}}}$, $\sigma_{\sigma_{\hat{r}_{ua,y,p}}}$ and, $\sigma_{\sigma_{\hat{r}_{ua,z,p}}}$ lead to the following pose-pairs out of 351 poses, where at least one of the three standard deviations is exemplary bigger than

$1m$: (1,3), (1,8), (1,9), (8,9), (13,14),(25,26),(24,27). A maximum of $223m$ in standard deviation was reached with the pose-pair (13,14).

The temporal changes over all m measurement points showed the following results for the *Equilibrium* method:

$$\begin{aligned} \mu_{\sigma_{\hat{f}_{ua}}} &= 0.54N, \quad \sigma_{\sigma_{\hat{f}_{ua}}} = 0.28N \\ \mu_{\sigma_{\hat{f}_{la}}} &= 0.19N, \quad \sigma_{\sigma_{\hat{f}_{ua}}} = 0.10N \end{aligned} \tag{7}$$

The spatial sensitivity analysis for the *Full* method revealed the following standard deviation measures for the 351 tested pose-pairs:

$$\begin{aligned} \mu_{\sigma_{\hat{f}_{ua}}} &= 3.9N, & \sigma_{\sigma_{\hat{f}_{ua}}} &= 1.2N \\ \mu_{\sigma_{\hat{r}_{ua,x}}} &= 59.7m, & \sigma_{\sigma_{\hat{r}_{ua,x}}} &= 100.3m \\ \mu_{\sigma_{\hat{r}_{ua,y}}} &= 28.2m, & \sigma_{\sigma_{\hat{r}_{ua,y}}} &= 46.9m \\ \mu_{\sigma_{\hat{r}_{ua,z}}} &= 40.2m, & \sigma_{\sigma_{\hat{r}_{ua,z}}} &= 56.6m \\ \mu_{\sigma_{\hat{f}_{la}}} &= 2.4N, & \sigma_{\sigma_{\hat{f}_{la}}} &= 0.8N \\ \mu_{\sigma_{\hat{r}_{la,x}}} &= 56.3m, & \sigma_{\sigma_{\hat{r}_{la,x}}} &= 92.6m \\ \mu_{\sigma_{\hat{r}_{la,y}}} &= 26.8m, & \sigma_{\sigma_{\hat{r}_{la,y}}} &= 43.9m \\ \mu_{\sigma_{\hat{r}_{la,z}}} &= 41.2m, & \sigma_{\sigma_{\hat{r}_{la,z}}} &= 53.7m \end{aligned} \tag{8}$$

The spatial sensitivity analysis of the *Equilibrium* method showed the following standard deviation measures:

$$\begin{aligned} \mu_{\sigma_{\hat{f}_{ua}}} &= 6.74N, \quad \sigma_{\sigma_{\hat{f}_{ua}}} = 2.47N \\ \mu_{\sigma_{\hat{f}_{la}}} &= 3.55N, \quad \sigma_{\sigma_{\hat{f}_{ua}}} = 1.05N \end{aligned} \tag{9}$$

To find the optimal estimation poses, the spatial sensitivity analysis was performed with $\delta_f = 4N$ and $\delta_r = 0.05m$ for the *Full* method and $\delta_f = 4N$ for the *Equilibrium* method. Offline analysis revealed that the pose-pairs 14 (P_1) and 20 (P_2) had the best algorithm result for the *Full* method ($\hat{f}_{err,14,20} = 10.3N$), while pose 14 ($P_1$) was the best estimation pose for the *Equilibrium* method ($\hat{f}_{err,14} = 44N$). These estimation poses were used to estimate arm weight in the subsequent arm weight compensation efficacy analysis with EMG.

To provide a reference to an estimation that utilizes measurements of the whole data set, we have estimated the parameters again by utilizing all the 27 poses. First, the mean of the estimated values for the parameters of all ten subjects is calculated (for all subjects independently). Then, the differences between the estimated values of the *Full* or *Equilibrium* methods and these mean values are calculated for each subject. The means of these differences over all subjects for the *Equilibrium* method are:

$$\begin{aligned} \hat{f}_{ua} - \overline{\hat{f}}_{ua} &= 1.4N \\ \hat{f}_{la} - \overline{\hat{f}}_{la} &= -0.6N \end{aligned} \tag{10}$$

The means of the differences over all subjects for the *Full* method are:

$$\begin{aligned} \hat{\boldsymbol{r}}_{ua} - \overline{\hat{\boldsymbol{r}}}_{ua} &= (-1.3m, 0.8m, -0.7m) \\ \hat{\boldsymbol{r}}_{la} - \overline{\hat{\boldsymbol{r}}}_{la} &= (-1.5m, 0.9m, -0.4m) \\ \hat{f}_{ua} - \overline{\hat{f}}_{ua} &= 1.6N \\ \hat{f}_{la} - \overline{\hat{f}}_{la} &= -2.4N. \end{aligned} \tag{11}$$

Additionally, the mean estimation results over 10 subjects' estimation results in the selected pose-pair (14,20) are:

$$\begin{aligned} \hat{\boldsymbol{r}}_{ua} &= (0.01m, -0.03m, -0.01m) \\ \hat{\boldsymbol{r}}_{la} &= (0.01m, -0.03m, -0.01m) \end{aligned} \tag{12}$$

The point of mass location vectors of the upper and lower arm are inside of the human body. The mean estimation results over 27 poses and 10 subjects are:

$$\begin{aligned} \overline{\hat{\boldsymbol{r}}}_{ua} &= (1.3m, -0.8m, 0.7m) \\ \overline{\hat{\boldsymbol{r}}}_{la} &= (1.4m, -0.9m, 0.4m). \end{aligned} \tag{13}$$

The point of mass distance vectors with coordinate system origin at the arm attachment cuffs $\overline{\hat{\boldsymbol{r}}}$ are clearly outside of the human body. The reason for this is when the estimation poses of a pose-pair are similar to each other, the mathematical calculations that are used for estimation get close to singularities. The effect of force sensors errors (noise and the drift) are reflected in the results more in these poses. Therefore, acquiring a lot of data in different poses and using all these data for the estimation is not always the best option. Care must be taken not to include any measurement data from these potentially singular poses or pose-pairs.

Author details

[1]Sensory-Motor Systems (SMS) Lab, Institute of Robotics and Intelligent Systems (IRIS), Department of Health Sciences and Technology (D-HEST), ETH Zurich, Switzerland and Reharobotics Group, Spinal Cord Injury Center, Balgrist University Hospital, Medical Faculty, University of Zurich, Switzerland, Lengghalde 5, 8092 Zurich, Switzerland. [2]Motor Learning and Neurorehabilitation Laboratory, ARTORG Center for Biomedical Engineering Research, University of Bern, Freiburgstrasse 3, 3010 Bern, Switzerland. [3]IAS-Lab, Department of Information Engineering, University of Padova, via Giovanni Gradenigo 6a, 35131 Padova, Italy. [4]Institute of Occupational Therapy, School of Health Professions, Zurich University of Applied Sciences, Technikumstrasse 81, 8401 Winterthur, Switzerland. [5]BIROMED-Lab, Department of Biomedical Engineering, University of Basel, Düsternbrooker Weg 20, 4123 Allschwil, Switzerland.

References

1. Gates, D.H., Walters, L.S., Cowley, J., Wilken, J.M., Resnik, L.: Range of motion requirements for upper-limb activities of daily living. American Journal of Occupational Therapy **70**(1) (2016). doi:10.5014/ajot.2016.015487
2. Holzbaur, K.R.S., Murray, W.M., Delp, S.L.: A model of the upper extremity for simulating musculoskeletal surgery and analyzing neuromuscular control. Annals of Biomedical Engineering **33**(6), 829–840 (2005). doi:10.1007/s10439-005-3320-7
